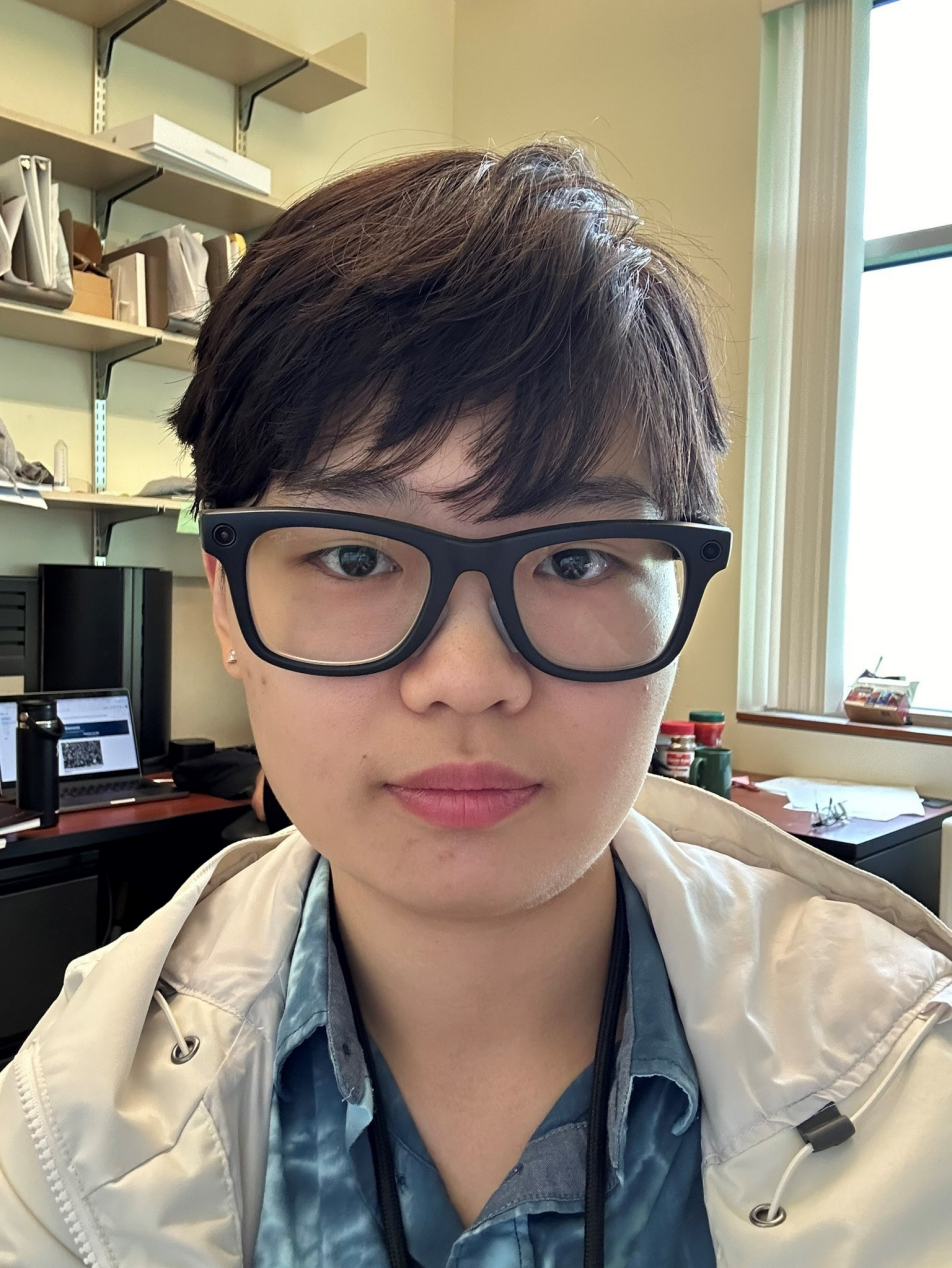# Mono‐/Bimetallic Doped and Heterostructure Engineering for Electrochemical Energy Applications

**DOI:** 10.1002/cssc.202401435

**Published:** 2024-11-05

**Authors:** Dawei Chu, Zhongwang Liang, Yi Cheng, Dong‐Feng Chai, Meijia Li

**Affiliations:** ^1^ Chemical and Biological Processing Group Pacific Northwest National Laboratory Richland, Washington 99354 United States; ^2^ College of Energy Engineering Huanghuai University Zhumadian 463000 China; ^3^ College of Chemistry and Chemical Engineering Qiqihar University Qiqihar 161006 China

**Keywords:** Monometallic-doped, Bimetallic-doped, Heterostructure, Electrochemical, Energy

## Abstract

Designing efficient materials is crucial to meeting specific requirements in various electrochemical energy applications. Mono‐/bimetallic doped and heterostructure engineering have attracted considerable research interest due to their unique functionalities and potential for electrochemical energy conversion and storage. However, addressing material imperfections such as low conductivity and poor active sites requires a strategic approach to design. This review explores the latest advancements in materials modified by mono‐/bimetallic doped and heterojunction strategies for electrochemical energy applications. It can be subdivided into three key points: (i) the regulatory mechanisms of metal doping and heterostructure engineering for materials; (ii) the preparation methods of materials with various engineering strategies; and (iii) the synergistic effects of two engineering approaches, further highlighting their applications in supercapacitors, alkaline ion batteries, and electrocatalysis. Finally, the review concludes with perspectives and recommendations for further research to advance these technologies.

## Graphical abstract

This review analyzed the latest advancements in mono‐/bimetallic doped and heterostructure engineering for various materials, including the preparation methods employed, factors affecting and synergistic effects, and highlighted their applications in supercapacitors, alkaline ion batteries, and electrocatalysis. Finally, the review concluded with perspectives and recommendations for further research to advance these technologies.

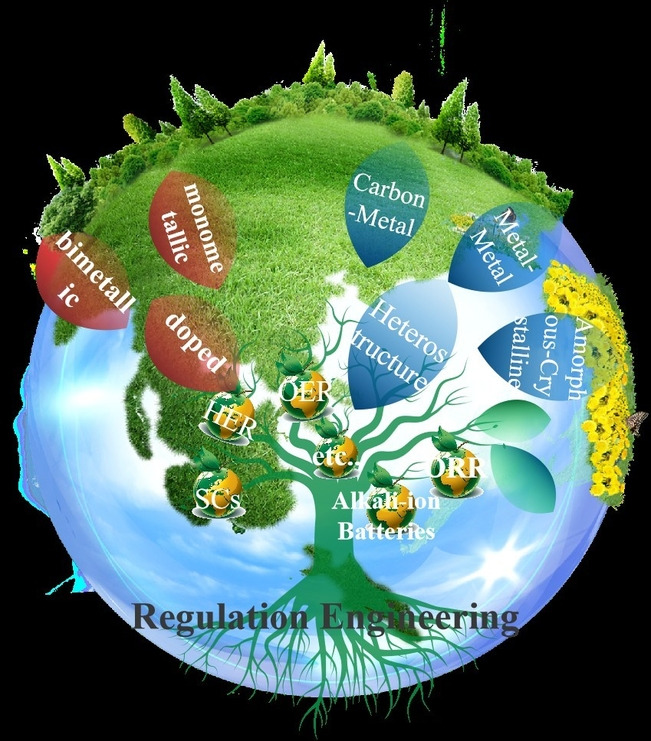

## 1 Introduction

According to Industry 4.0, human lifestyles are increasingly dominated by the fourth industrial revolution, which necessitates a robust energy supply.^[1]^ However, the majority of energy is currently sourced from oil, coal, and natural gas, leading to environmental degradation.^[6]^ Therefore, there is an urgent need to explore and develop these novel technologies and renewable energy sources that can pragmatically compete with traditional fossil fuels in the future. Supercapacitors (SCs), alkaline ion batteries, and electrocatalytic processes like the hydrogen evolution reaction (HER), oxygen evolution reaction (OER), and oxygen reduction reaction (ORR) are considered highly efficient technologies for electrochemical energy conversion and storage.^[11]^ These technologies convert energy into chemical or electrical forms and store or release it with minimal pollution using redox electrochemistry. SCs and batteries store electric energy from the grid for electronic devices. HER and OER are vital for water splitting, producing hydrogen and oxygen for fuel cells and rechargeable metal‐O_2_ batteries *via* HOR and ORR.^[18]^

Researchers are exploring highly efficient materials using various strategies such as structure, defect, and alloying engineering to address electrode weaknesses in electrochemical energy applications.^[25]^ However, many materials fail to meet specific requirements for different electronic devices and storage technologies. Electronic structure plays a pivotal role in determining physical and chemical properties.^[30]^ Therefore, manipulating the electronic structure of materials is crucial for achieving optimized properties and desired functionalities. Currently, electronic structures of designed materials can be systematically adjusted through metal doping and heterostructure engineering. So far, significant breakthroughs have been achieved in the development of mono‐/bimetallic doped and heterostructure materials using a series of methods.^[36]^ Numerous materials, such as Co‐ and Fe‐doped MoS_2_/polypyrrole, Fe/Co/Ni/Cu/Zn‐doped ReS_2_, Ni‐doped Mn_3_O_4_, Ru‐doped Ni/Fe MIL‐53 MOF, and Ni nanoparticles‐Ni_3_C heterostructures, have been successfully synthesized for various applications.^[39]^ Researchers have utilized advanced instrumentation to characterize the morphology, crystal structures, surface chemical properties, and electrochemical performances of these samples.^[44]^ Researchers use ex‐situ techniques such as X‐ray photoelectron spectroscopy (XPS) and synchrotron‐based X‐ray absorption near‐edge spectroscopy (XANES) to explore the origins of high intrinsic activity and electronic interaction changes in these materials.^[48]^ Furthermore, density functional theory (DFT) calculations are also employed to investigate activity origins and mechanisms.^[51]^

**Figure 1:**
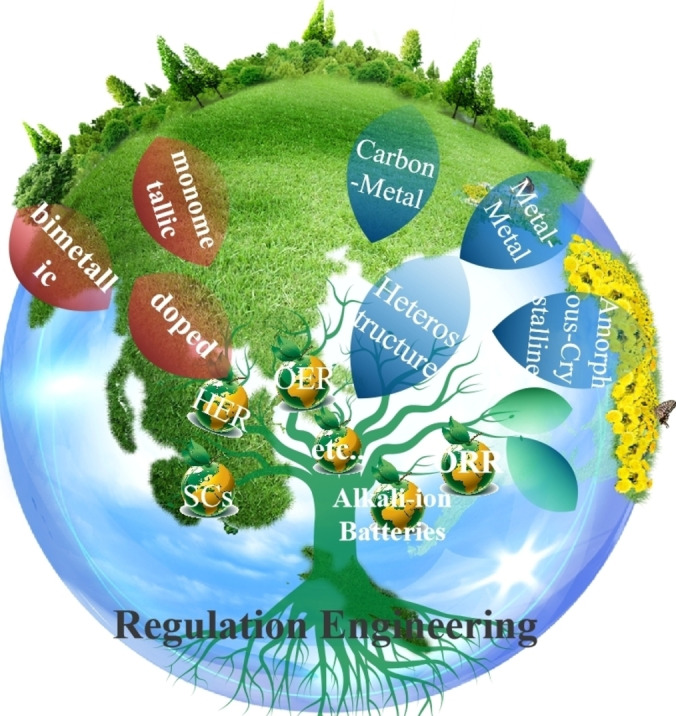
Schematic diagram of the topics covered in this review.

The following sections provide a comprehensive overview of the latest research progress in material design through metal‐doped and heterogeneous‐interface engineering for electrochemical energy conversion and storage (Figure 1). The review begins by summarizing well‐established synthetic methods for these two strategies. It then discusses various types of metal doping (e. g., Fe, Co, Ni) and heterostructures, including amorphous‐crystalline, carbon‐metal, and metal‐metal based heterointerfaces. Moreover, the review details advancements in these strategies for applications in supercapacitors, rechargeable batteries, and electrocatalysis.

## 2 Mono‐/Bimetallic Doped Engineering

**Figure 2:**
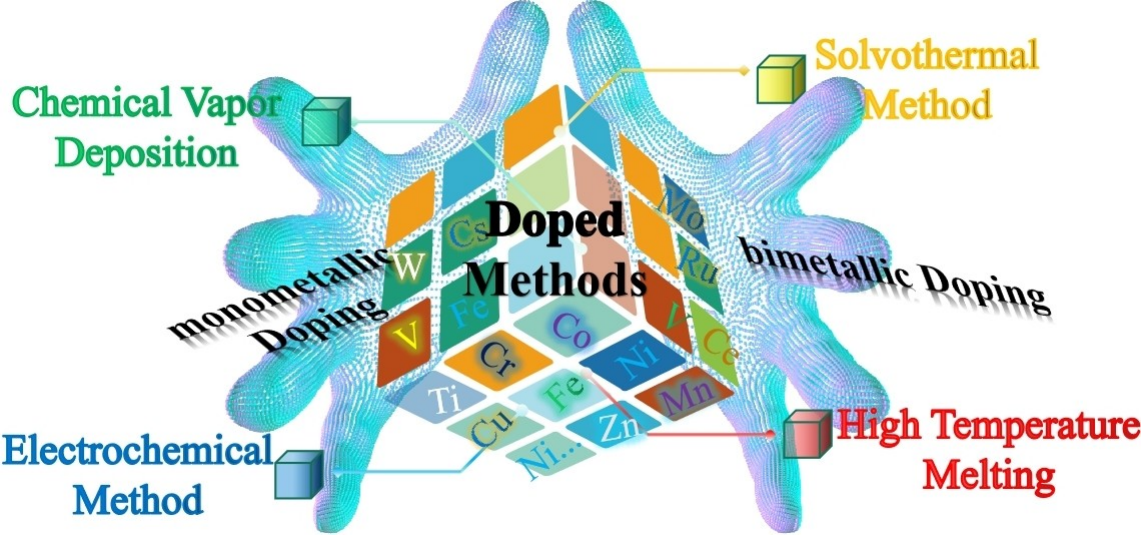
Schematic diagram of the topics covered in this following section.

The synthesis of exceptional materials often involves mono‐ or bimetallic doped strategies.^[53]^ Metal doping has emerged as a crucial approach that can fundamentally alter the electronic or ionic states of active materials for electrochemical energy storage.^[56]^ Furthermore, developing controllable doping methods is essential to achieving optimized performance and warrants further advancement. This section will delve into detailed discussions on the various classifications, synthetic techniques, characterization methods and the regulatory mechanisms of mono‐ or bimetallic doped engineering (Figure 2).

### 2.1 Monometallic Doping Engineering

Metal dopants (such as Ni, Co, Mn, Fe, Cr, Mo, Ti, Ru, Zr, etc.) have proven to be highly effective strategies for addressing issues such as low conductivity, cycling life, and structural stability in electrochemical energy applications.^[58]^ To date, various monometallic‐doped materials have been synthesized using several main methods, including solvothermal/electrochemical methods, chemical vapor deposition (CVD), and high‐temperature melting.^[64]^ Iron (Fe) is highly versatile in altering the electronic structure of base catalysts due to its abundant d‐band electrons.^[66]^ Enhanced performance from Fe‐doping strategy generally stems from two main mechanisms: i) altering the local electronic structure and ii) boosting the electrical conductivity of the catalysts.^[68]^

**Figure 3:**
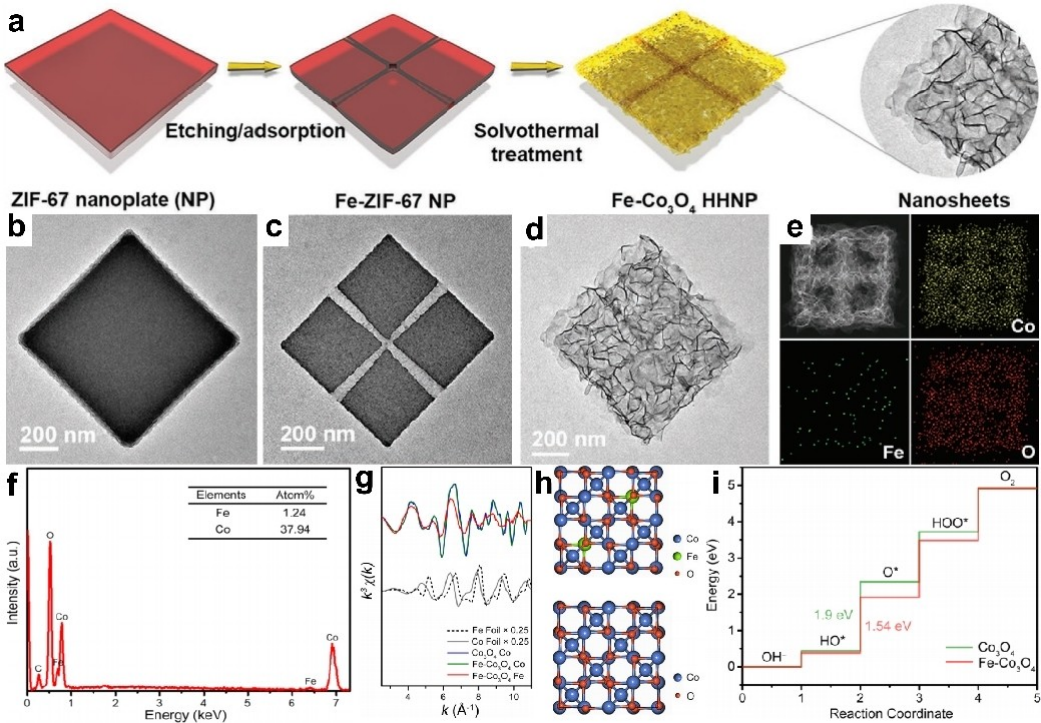
(a) Schematic illustration of cross‐channeled Fe‐Co_3_O_4_ HHNPs. TEM of (b) ZIF‐67 NPs, (c) Fe‐Co_3_O_4_ HHNPs. (e) HAADF‐STEM image and elemental mapping images of signal Fe‐Co_3_O_4_ HHNP. (f) EDX analysis of cross‐channeled Fe‐Co_3_O_4_ HHNPs. (g) EXAFS spectra of the Fe‐Co_3_O_4_ HHNPs. The optimized model of Fe‐Co_3_O_4_: (h) Crystal model of Co_3_O_4_. (i) Free energy diagram for the OER process over Co sites. Reproduced with permission from ref.^[70]^ Copyright 2020, WILEY‐VCH Verlag GmbH & Co. KGaA, Weinheim.

**Figure 4:**
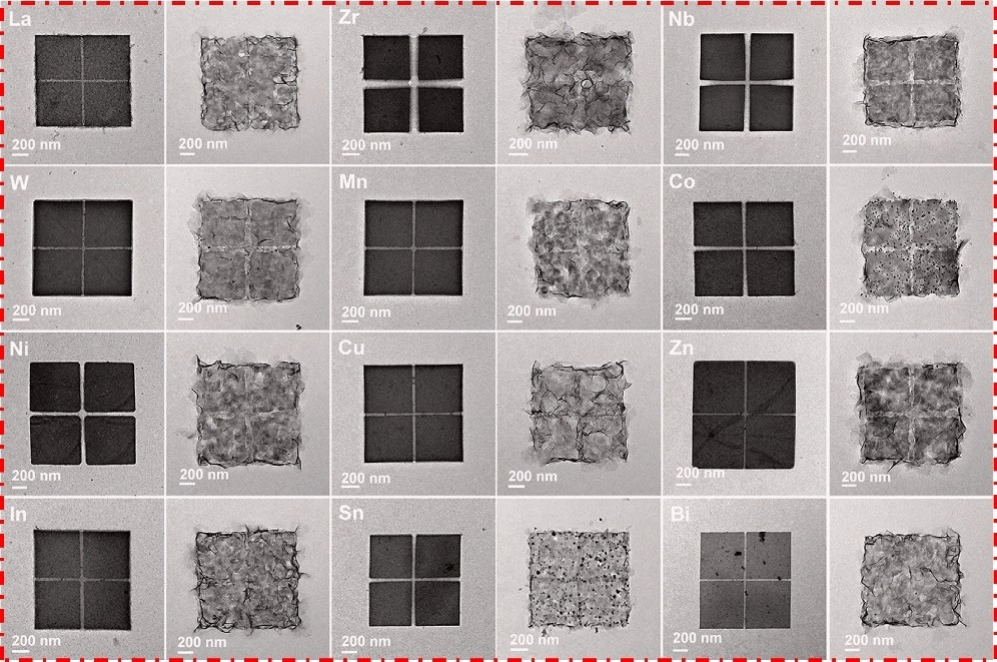
TEM images of M‐ZIF‐67 NPs (left) and M–Co_3_O_4_ HHNPs (right) of 12 metal elements. Reproduced with permission. Reproduced with permission from ref.^[70]^ Copyright 2020, WILEY‐VCH Verlag GmbH & Co. KGaA, Weinheim.

Lou et al. conducted a notable study involving the synthesis of Fe‐doped Co_3_O_4_ hierarchically hollow nanoplates (Fe‐Co_3_O_4_ HHNPs) from dual metal salts, starting with zeolitic imidazolate framework‐67 (ZIF‐67) NPs and Fe‐ZIF‐67 NPs (Figure 3c).^[70]^ They confirmed the presence of Fe dopants using techniques like Energy‐dispersive X‐ray (EDX) spectroscopy (Fe to Co molar ratio approximately 1 : 30.6) and extended X‐ray absorption fine structure (EXAFS) measurements (Figure 3f, g). Density functional theory (DFT) results (Figsure 3h–j) suggests that surface‐bound Fe atoms significantly boosted electrocatalytic activity towards the oxygen evolution reaction (OER) by lowering energy differences in adsorption from OH^*^ to O^*^. Additionally, the study summarizes the incorporation of 13 metal dopants into ultrathin Co_3_O_4_ nanosheets (Figure 4), highlighting Fe−Co_3_O_4_ and other transition metal‐decorated catalysts as promising for OER (262 mV at 10 mA cm^−2^).

**Figure 5:**
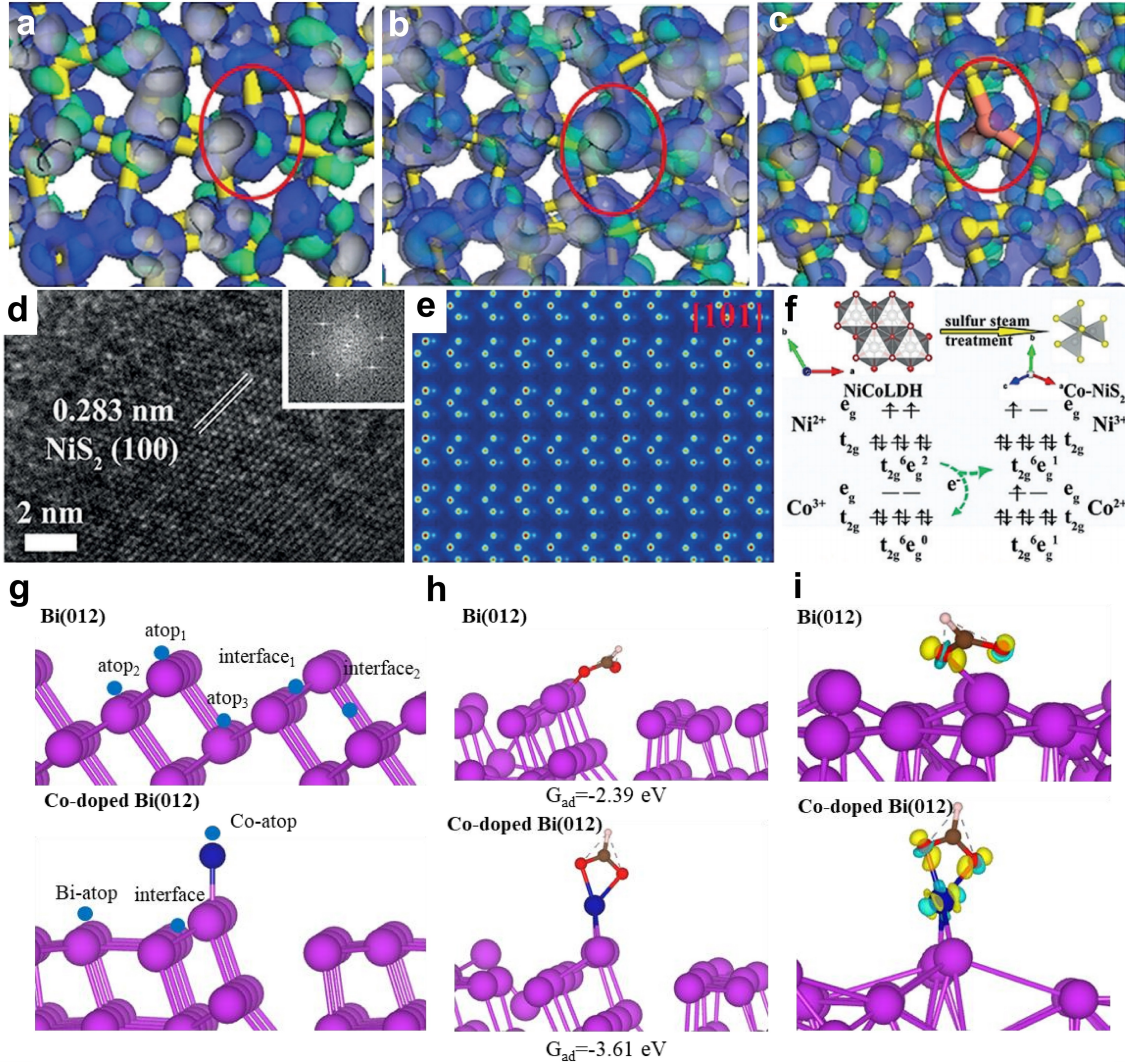
The real spatial contour plots for bonding and anti‐bonding orbitals near EF for (a) Co−NiS_2_ NSs, (b) Fe−NiS_2_ NSs, and (c) Cu−NiS_2_ NSs. The red ovals of (a)–(c) represent the different doping metals as Co, Fe, and Cu in NiS_2_. (d) HRTEM images of Co−NiS_2_ NSs. Inset: the atomic arrangement FFT image of Co−NiS_2_ NSs on (100) face. (e) The surface intensity and atomic columns simulated by using QSTEM software along [101]. (f) The characterization of structure and electron change after Co‐doping. Reproduced with permission. Copyright 2019, Wiley‐VCH. Reproduced with permission from ref.^[71]^ Copyright 2019, WILEY‐VCH Verlag GmbH & Co. KGaA, Weinheim. (g) The configurations for Bi(012) and Co‐doped Bi(012). (h) The calculated free energy of OCHO^*^ adsorption over Bi(012) and Co‐doped Bi(012). (i) Differential charge density for OCHO^*^ over Bi(012) and Co‐doped Bi(012). The brown, red, white, purple, blue sphere and yellow or blue areas represent C, O and H, Bi, Co and a gain or loss of electrons, respectively. Reproduced with permission from ref.^[72]^ Copyright 2024, WILEY‐VCH Verlag GmbH & Co. KGaA, Weinheim.

Recent research highlights the critical role of e_g_ electrons in catalytic activity, as they orient favorably towards surface‐bound intermediates and influence the energetics of electrochemical reactions. Xi′s group developed Co, Fe, Cu−NiS_2_ nanosheets (NSs) using a novel modulation strategy to optimize electronic configurations and atomic arrangements flexibly.^[71]^ They details the doping of Co (Figure 5a), Fe (Figure 5b), and Cu (Figure 5c), targeting surface bonding and anti‐bonding orbitals near the Fermi level (EF). High‐resolution TEM (HRTEM), FFT image analysis (inset of Figure 5d), and QSTEM (Figure 5e) confirmed atomic rearrangement due to successful Co doping in NiS_2_, altering its standard atomic arrangement on the (100) face. The authors characterize electron changes post‐Co doping (Figure 5f), noting that Co‐NiS_2_ NSs with Ni^3+^ and Co^2+^ exhibited a t_2g_^6^e_g_^1^ configuration, distinct from Ni^2+^ and Co^3+^ in NiCo−LDH. Co−NiS_2_ NSs shows superior HER performance with an overpotential of 80 mV at 10 mA cm^−2^ in alkaline media. Co dopants shifted Ni‐3d bands near EF, enhancing electron‐transfer capability for nearly barrier‐free electron transfer in HER under alkaline conditions. Co dopants can moderate H‐bonding energy for HER catalysts, even in trace amounts, and effectively alter the electronic structure. This highlighting the importance of optimal Co doping levels in enhancing intrinsic conductivity and reducing ΔG_H*_.

Wang et al. synthesized metal (Co/Ni/Fe/Cu)‐doped bismuth nanosheet catalysts (BOON) to enhance electrochemical CO_2_ reduction to form electrolyte‐free formic acid.^[72]^ They demonstrates that Co_0.05_‐doped BOON achieved ~90 % HCOO‐ selectivity and a low overpotential of ~1.0 V at 200 mA cm^−2^. These results are attributed to Co doping in Bi nanosheets, which effectively enhancing the electron density of Bi p‐orbitals and their delocalization, shifting them closer to the Fermi level. Figures 5g and f investigate the role of the Co element in CO_2_RR to formic acid. Clearly, OCHO^*^ shows higher priority for adsorption on the Co site of Co‐doped Bi(012) (−3.61 eV) compared to pure Bi(012) (−2.93 eV). This indicates that the Co‐doped Bi(012) surface has superior binding affinity for OCHO^*^ than pure Bi, with most oxygen atoms from OCHO^*^ binding to Co atoms rather than just one oxygen atom binding to pure Bi(012). Therefore, the metal‐doped strategy can enhance catalyst performance by tuning surface adsorption affinity and reactivity.

Transition metal d‐orbitals in the outermost layer are sensitive to external factors like crystal fields and coordination environments. In contrast, lanthanide (Ln) elements have buried 4 f orbitals shielded by 5 s/p orbitals, reducing external influences and allowing precise tuning of metal‐other atom bonding for superior electrochemical performance. While these metal dopants, unlike Fe, Co, Ni, etc., have not received extensive study, recent reports highlight their effectiveness in enhancing performance. Further research is required to fully elucidate their operational mechanisms.

**Figure 6:**
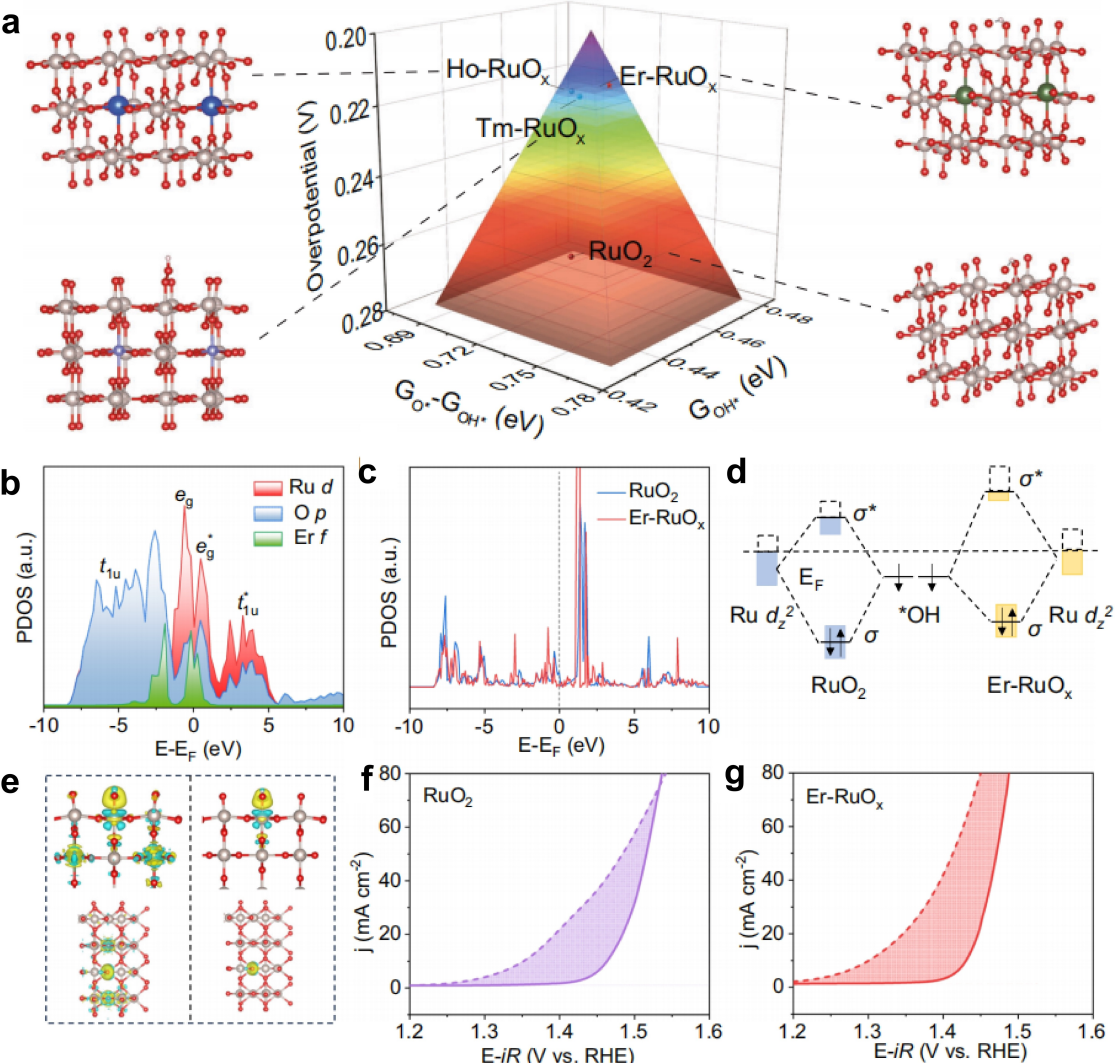
(a) The volcano plot for Ln group‐doped catalysts and relative structures based on the DFT. (b) The calculated PDOS of different elements within Er‐RuO_x_. (c) The PDOS of Ru d_z_^2^ orbital of various catalysts. (d) Schematic diagram of orbital hybridization between d_z_^2^ orbital of RuO_2_ and Er‐RuO_x_ and the ^*^OH bonding orbitals. (e) Charge density difference of ^*^OH‐adsorbed Er‐RuO_x_ (left) and RuO_2_ (right). Yellow and blue iso‐surfaces represent charge accumulation and depletion, respectively. Polarization curves of (f) RuO_2_ and (g) Er‐RuO_x_ in 0.5 M H_2_SO_4_ solution with (dashed lines) and without (solid lines) 1 M methanol. Reproduced with permission from ref.^[73]^ Copyright 2024, Springer Nature.

Recently, Guo′s group first modulated the electronic configurations of Ru−O by introducing Er elements to investigate the shielding effect of 5 s/5p orbitals.^[73]^ This work initially presented the overpotential of Ln group‐doped catalysts to illustrate the free energy between ^*^O and ^*^OH using DFT calculations (Figure 6a). Er‐RuO_x_ achieves superior ^*^OH binding strength and optimal free energy, resulting in a lower theoretical overpotential. Based on the theoretical and experimental findings (Figures 6b–g), Er atoms slightly alter the electronic environment of Ru d‐orbitals, thereby lowering the d‐band center of Ru. This facilitates stronger electron transfer between ^*^OH and Er−O−Ru compared to pure RuO_x_, demonstrating enhanced ^*^OH adsorption. Similarly, Er−RuO_x_ exhibits a greater current difference between the MOR and OER compared to RuO_2_ due to precise changes in Ru−O co‐valency through d‐p‐f orbital hybridization involving Ln elements. Ln elements provide a subtle means to slightly regulate the electrochemical performance of catalysts based on abundant electronic energy levels, different 4 f‐orbital electronic structures, shielding effects of 5 s/5p orbitals, and the capability to adapt to various coordination environments.

### 2.2 Bimetallic Doping Engineering

Monometallic doping has been proven effective in enhancing catalytic activity.^[74]^ However, the extent of this increase is often limited due to insufficient regulation of the electronic structure. It has been noted that the electronic interactions between elements in monometallic‐doped materials are generally overlooked. This raises the question of whether the lattice and electronic structures could be more effectively controlled by simultaneously doping multiple metals to further enhance catalytic performance.^[75]^ Bimetallic doping has garnered significant research interest as a method to achieve better regulation of the electronic structure.^[76]^ It has been proposed that the synergistic coupling effect of dual doping enhances overall performance.^[77]^

**Figure 7:**
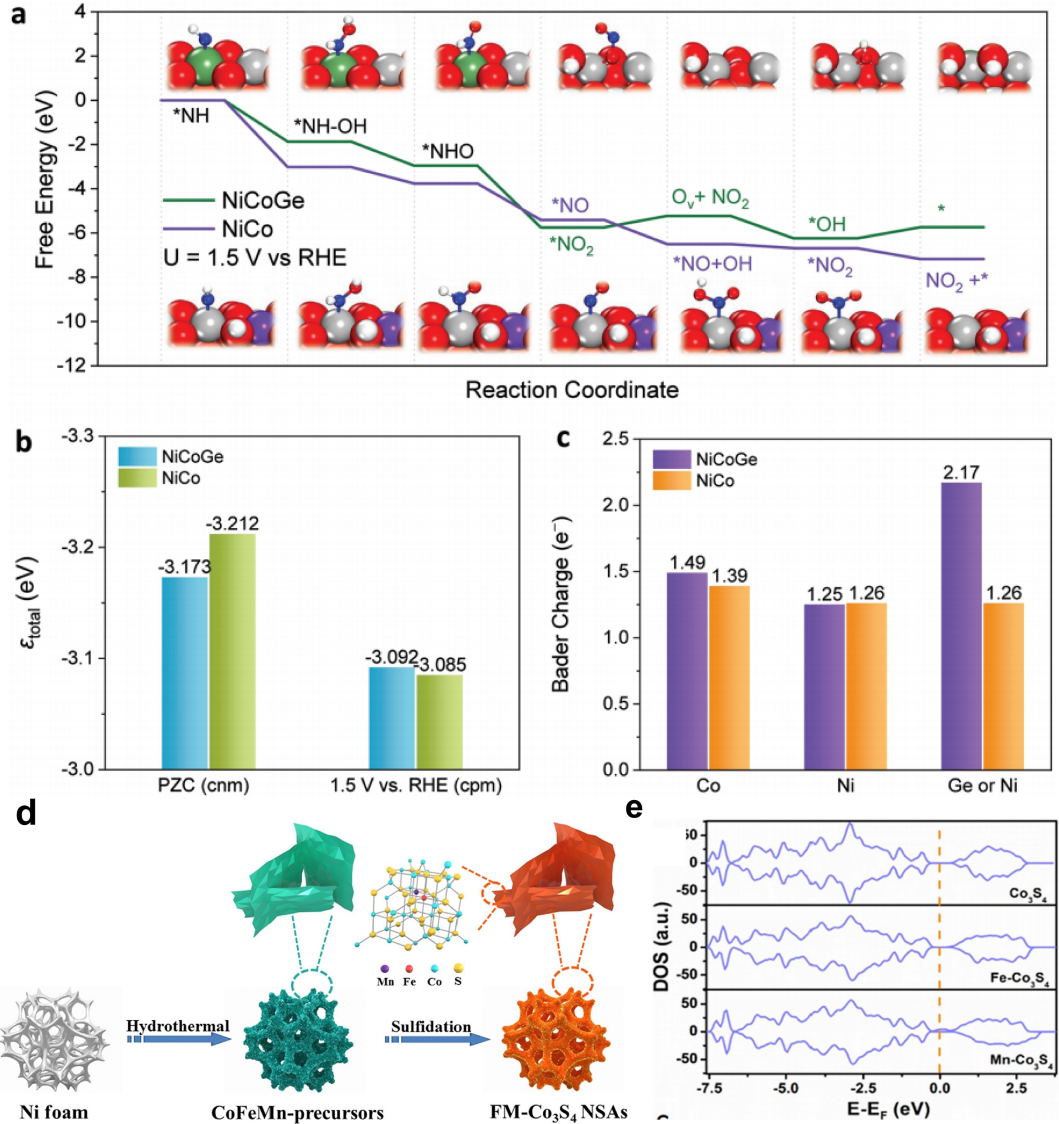
(a) Free energy diagram for ^*^NH to NO_2_^−^. (b) Total band centre for NiCo and NiCoGe. c) Analyses for Bader charge of adjacent Co, Ni, and Ge/Ni at the first layer at U=1.5 V_RHE_. The grey, purple, green, brown, red, white, and blue spheres represent Ni, Co, Ge, C, O, H, and N atoms, respectively. Reproduced with permission. Copyright 2023, Wiley‐VCH. (d) Schematic illustration of the synthetic procedures of FM‐Co_3_S_4_ ultrathin NSAs. (e) The total DOS of different samples. Reproduced with permission from ref.^[81]^ Copyright 2021, Elsevier B.V.

Metal dopants can generate oxygen vacancies around the surfaces of host materials, thereby enhancing their electronic structure and modulating bulk electronic properties to improve electrical conduction.^[79]^ For instance, Shi‐zhang Qiao designed germanium (Ge) and cobalt (Co) co‐doped Ni‐based materials (NiCoGe), achieving a notable Faradaic efficiency of 84.9 % at 1.4 V.^[80]^ The presence of Ge and Co tailors the electronic structure of Ni(OH)_2_, facilitating the transformation of urea to NO_2_^−^. According to Figures 7a–c, the active sites of NiCoGe shift from Ni atoms to O atoms during this transformation. Consequently, Ge atoms accelerate NO_2_^−^ generation by forming NO combinations with adjacent O atoms due to weaker urea adsorption on Ge sites. This transformation of active sites results from differing electronic properties between NiCoGe and NiCo. Ge atoms fail to retain more electrons during *NO adsorption, leading to linkage with adjacent O atoms and altering the original reaction pathway. Furthermore, incorporating Fe ions enhances the specific capacity of final electrodes, while Mn ions contribute to excellent rate capabilities in reversible redox processes. Hu and colleagues developed a robust electrode based on Fe and Mn co‐doped Co_3_S_4_ (FM−Co_3_S_4_) ultrathin nanosheet arrays (NSAs) on Ni foam *via* a hydrothermal method (Figure 7d).^[81]^ Benefiting from synergistic effects of different transition metal ions, FM‐Co_3_S_4_ ultrathin NSAs exhibits a high specific capacity of 390 mAh g^−1^ at 5 A g^−1^. Density functional theory (DFT) calculations confirms that Mn doping significantly reduces the energy gap of Co_3_S_4_, favorably impacting electrochemical performance (Figure 7e). Other dual metal dopants, such as Mn−Ni, efficiently optimize electron structures by decreasing e_g_ orbital electron numbers and adjusting the O 2p‐band closer to the Fermi level, thereby enhancing bifunctional activity. Based on these examples, metal doping lowers overpotential, enhances durability and rate performance of materials by modulating hydrogen adsorption free energy, altering local electronic structures, especially around active centers, and increasing electrical conductivity.

### 2.3 Heterostructure Engineering

**Figure 8:**
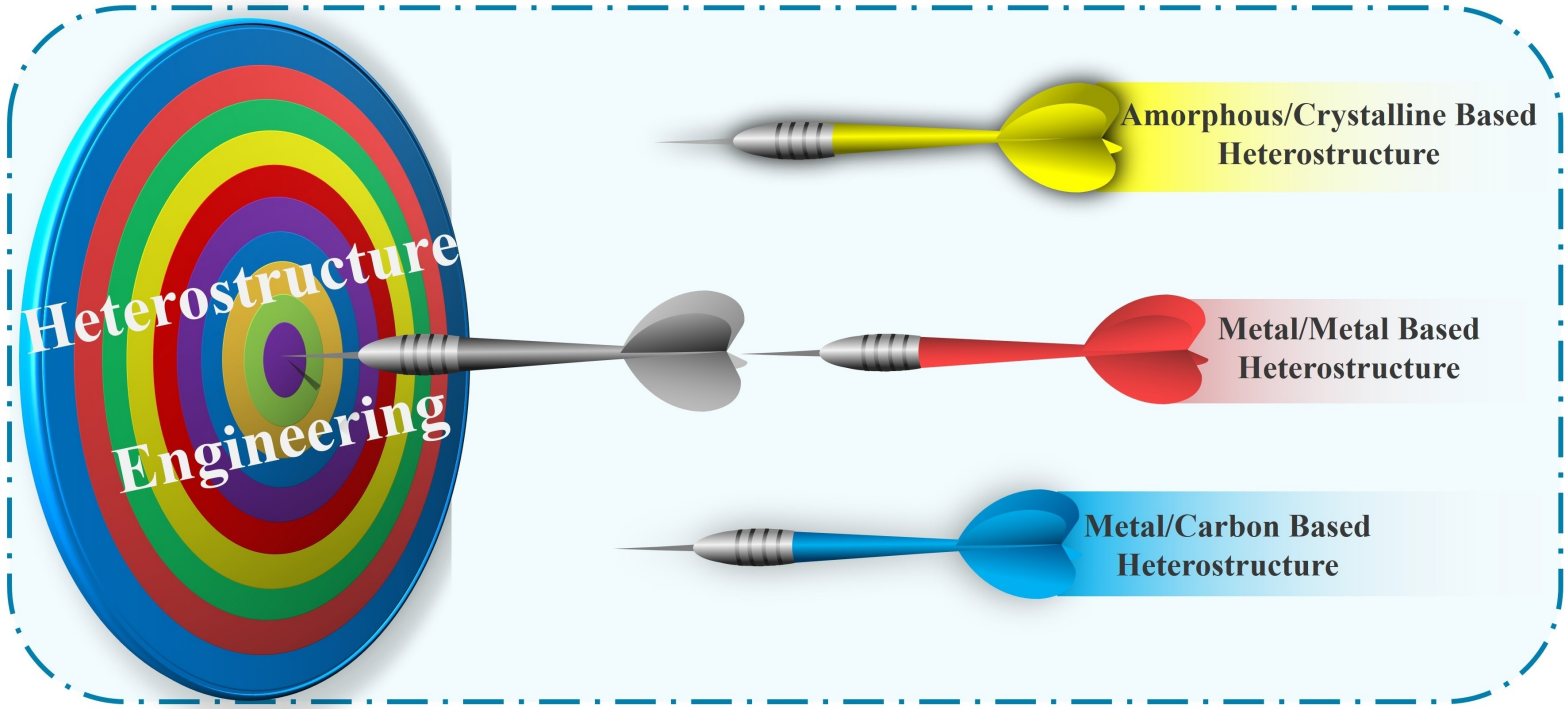
Schematic diagram of the topics covered in this following section.

Significant advancements have been made in improving materials’ reaction kinetics through the construction of heterostructures.^[82]^ These structures not only shorten ion diffusion paths but also generate a built‐in electric field at heterointerfaces, thereby further enhancing electrochemical reaction kinetics.^[83]^ Various synthesis methods have enabled researchers to design specific heterostructure configurations, including amorphous/crystalline,^[84]^ metal‐metal,^[85]^ metal‐carbon,^[86]^ carbon‐carbon,^[87]^ and conducting polymer‐carbon heterostructures.^[88]^ This review will systematically present the following heterogeneous structures (Figure 8).

### 2.4 Amorphous/Crystalline‐Based Heterostructure

**Figure 9:**
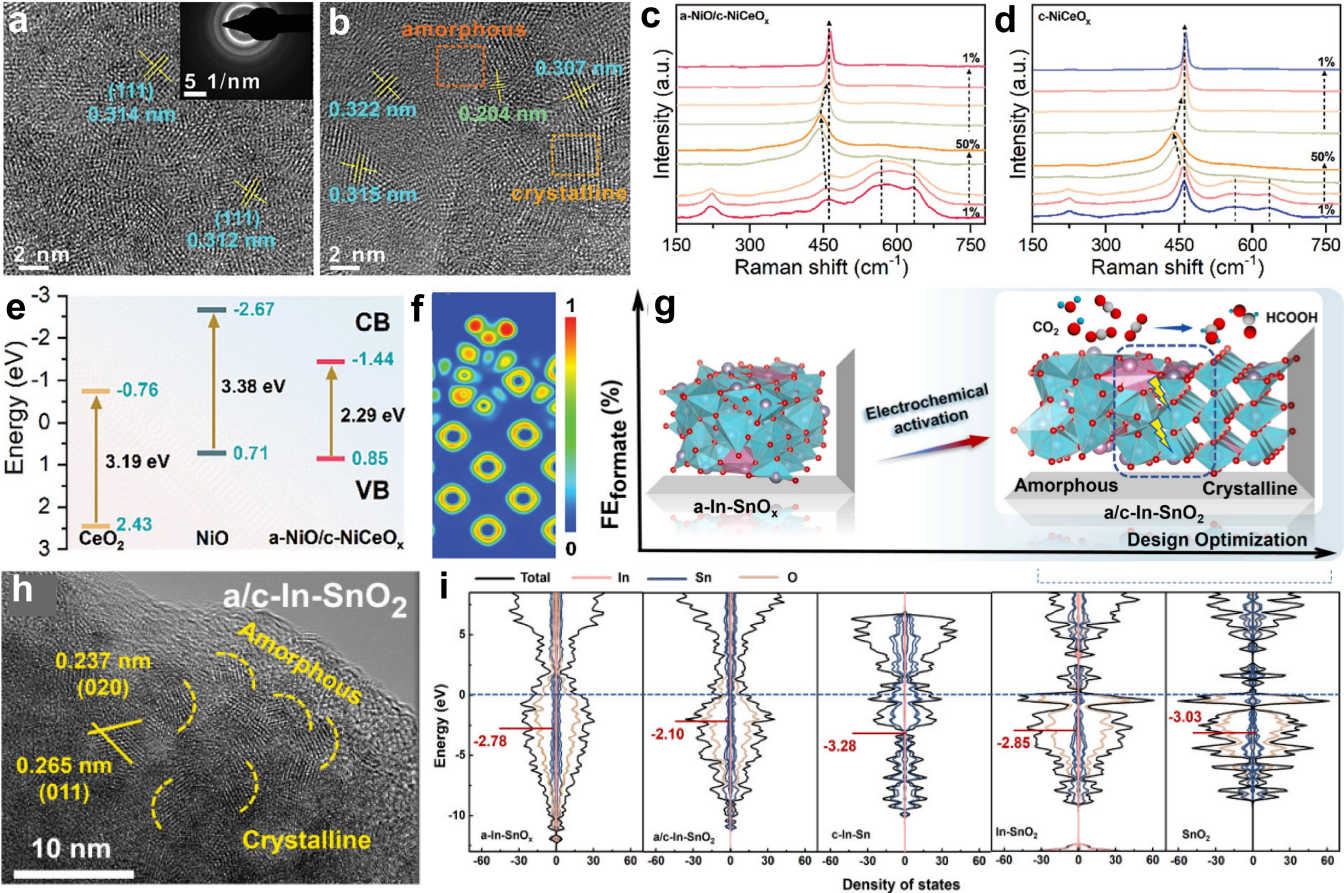
(a)–(b) HRTEM images of a‐NiO/c‐NiCeO_x_ with amorphous/crystalline heterostructure. (c)–(d) Raman spectra of amorphous‐NiO/crystalline‐NiCeO_x_ and crystalline‐NiCeO_x_. (e) Energy band structures of various samples. (f) The electron localization function map of amorphous‐NiO/crystalline‐NiCeO_x_. Reproduced with permission from ref.^[91]^ Copyright 2024, Wiley‐VCH. (g) Schematic illustration of the formation process of a/c‐In‐SnO_2_. (h) HRTEM images of a/c‐In‐SnO_2_. (i) Projected DOS of different samples. Reproduced with permission from ref.^[92]^ Copyright 2024, WILEY‐VCH Verlag GmbH & Co. KGaA, Weinheim.

Amorphous materials naturally contain numerous defects that serve as active sites during electrocatalysis.^[89]^ However, their low electrical conductivity severely limits charge transfer kinetics.^[90]^ Therefore, achieving a balanced composition of amorphous and crystalline phases is crucial for designing effective materials for electrochemical energy applications. For example, Tang et al. developed an amorphous‐NiO/crystalline‐NiCeO_x_ (a‐NiO/c‐NiCeO_x_) heterostructure using a coordination‐assisted approach (Figures 9a and b).^[91]^ This hybrid structure exhibited low overpotentials and high catalytic stability for the OER due to the heterogeneous amorphous‐crystalline interface. It enhances charge transfer, modifies local electron density, and facilitates adsorption between oxygen‐based intermediates and Ni sites, thereby reducing dissociation‐related energy barriers (Figures 9c and d). Valence‐band (VB) X‐ray Photoelectron Spectroscopy (XPS) analysis indicated that the electronic conductivity of c‐NiCeO_x_ was lower compared to a‐NiO/c‐NiCeO_x_, suggesting higher electron migration rates in the latter (Figure 9e and f). The formation of an amorphous/crystalline heterostructure increases the exposure of active sites, improving charge transfer compared to fully crystalline or amorphous counterparts. Considering that amorphous materials undergo unavoidable and uncontrollable reconstruction during electrocatalysis, Deng′s Group designed amorphous In‐doped SnO_x_ (a‐In‐SnO_x_) for in‐situ reconstruction of highly active structures (a/c‐In‐SnO_x_) *via* electrochemical activation during CO_2_ Reduction Reaction (CO_2_RR), resulting in an in‐situ amorphous‐crystalline interface (Figures 9g and h).^[92]^ This approach dynamically monitored catalytic reconstruction to verify practical active sites. Utilizing a‐In‐SnO_x_ to form a/c‐In‐SnO_2_, the p‐band center shifted significantly to −2.10 eV due to the unique amorphous‐crystalline electronic structure. Density of States (DOS) calculations supported these findings (Figure 9g). These results confirm that in‐situ reconstruction facilitates charge transfer during the electrochemical process, thereby enhancing selectivity for CO_2_RR.

### 2.5 Metal/Metal‐Based Heterostructure

**Figure 10:**
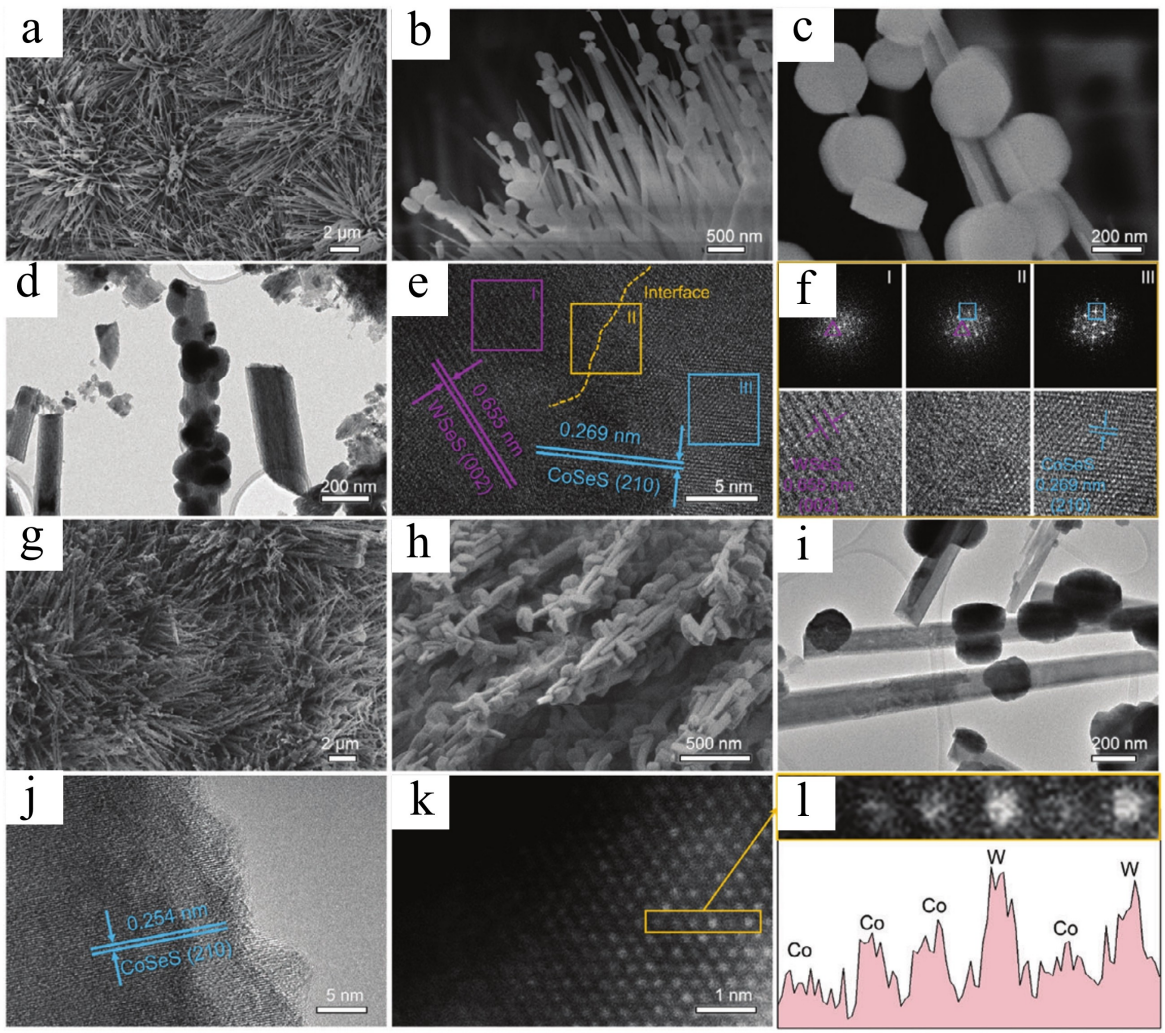
Morphological characterization ofthe formed a–f) WSeS/CoSeS NAs and g–l) W‐CoSeS NAs. (a–c) SEM images, (d) TEM image, (e) HRTEM image, (f) FFT patterns and the corresponding HRTEM images ofselected areas marked in (e). (g), (h) SEM images, (i) TEM image, (j) HRTEM image, (k) Cs‐HAADF‐STEM image, (l) magnified image ofmarked region in (k) and corresponding intensity profile. Reproduced with permission from ref.^[93]^ Copyright 2024, WILEY‐VCH Verlag GmbH & Co. KGaA, Weinheim.

Hybrids using different metal electrocatalysts can be extensively created through heterostructure engineering, enabling control over electron transfer, active sites, and catalytic activity by constructing coupling interfaces and leveraging the synergistic effects of heterostructures. For example, various heterostructures like Co/Co_9_S_8_ (as demonstrated by Qin et al.^[93]^), WSeS/CoSeS (as demonstrated by Bai et al.^[94]^), CoP@Ni_2_P,^[95]^ CoS_1.097_/Ni_3_S_2_,^[96]^ CoPS_3_/CoS_2_,^[97]^ have been synthesized extensively to enhance properties. Metal‐metal based heterostructure interfaces represent an effective strategy for concurrently adjusting the electronic structure and active sites of catalysts. These characteristics not only retain the intrinsic material activities of each component but also confer novel or improved performances due to the synergistic effects at the interface. Qin et al. successfully designed and realized well‐ordered 3D‐quaternary WSeS/CoSeS heterojunction nanoarrays and high‐valence W‐doped CoSeS nanoarrays with a “nanoparticles‐onto‐nanowire” structure (abbreviated as WSeS/CoSeS NAs and W‐CoSeS NAs, respectively), which were employed as self‐supported HER and OER electrocatalysts.^[93]^ SEM and TEM measurements were conducted to observe the size and morphology of WSeS/CoSeS NAs and W‐CoSeS NAs (Figure 10). As observed in the SEM images, the uniform “nanoparticles‐onto‐nanowire” structure of WSeS/CoSeS NAs (Figures 10a–c) and W‐CoSeS NAs fully covers the Ni foam (Figures 10g, h). The rotating homogeneous hydrothermal reaction ensures the quasi‐hexagonal prism morphology of the particles. During the doping process, Co^2+^ ions partially dissolve into the solution and coprecipitate with W species on the surface of the CCH NAs.^[98]^ Upon enlargement of the local zone in Figures 10d–i by TEM, small nanoparticles with diameters of approximately 200 nm were tightly confined onto the nanoarrays.

**Figure 11:**
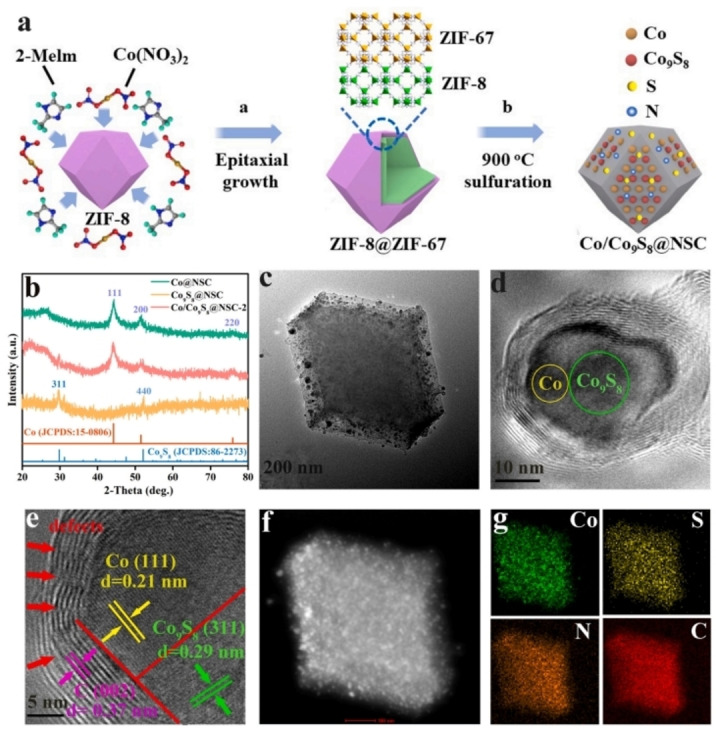
(a) Schematic diagram of the synthesis of Co/Co_9_S_8_@NSC catalyst. (b) XRD patterns of the Co@NSC, Co_9_S_8_@NSC and Co/Co_9_S_8_@NSC‐2. (c) TEM image, (d‐e) HRTEM images, (f) HAADF‐STEM image and (g) elemental mapping analysis of as‐obtained Co/Co_9_S_8_@NSC‐2. Reproduced with permission from ref.^[94]^ Copyright 2024, Elsevier B.V.

The structure of WSeS/CoSeS nanostructures was analyzed using high‐resolution transmission electron microscopy (HRTEM), as shown in Figure 10e. Clear hetero‐interfaces between WSeS and CoSeS components were observed (dashed line). Lattice fringes with spacings of 0.655 nm (WSeS (002) facets) and 0.269 nm (CoSeS (210) facets) were identified. Fast Fourier transform (FFT) diffraction patterns at adjacent WSeS and CoSeS domains confirmed the formation of heterointerfaces (Figure 10f). In W‐CoSeS nanocomposites, CoSeS structure was discernible from lattice fringes in HRTEM (Figure 10j). Cs‐corrected high‐angle annular dark‐field scanning transmission electron microscopy (Cs‐HAADF‐STEM) validated W doping in CoSeS, showing dense bright spots (Figure 10k, l). This study details a method for synthesizing self‐supported “nanoparticles‐onto‐nanowire” structured WSeS/CoSeS NAs and W‐CoSeS NAs, exhibiting excellent catalytic reactivity for HER and OER, respectively. Bai et al. developed Co/Co_9_S_8_ heterojunctions anchored on N, S co‐doped carbon materials via partial vulcanization‐pyrolysis.^[94]^ The polyhedral Co/Co_9_S_8_@NSC catalyst was synthesized in two steps (Figure 11a) using a thermostatic reduction strategy. Thiourea controlled sulfide‐triggered calcination, regulating Co/Co_9_S_8_ heterostructure formation. X‐ray diffraction (XRD) patterns showed crystalline Co@NSC, Co_9_S_8_@NSC, and various Co/Co_9_S_8_@NSC phases by adjusting Co/S ratio (Figure 11).

In Figure 11b, diffraction peaks at 44.2°, 51.5°, and 75.9° correspond to the (111), (200), and (220) crystal planes of Co in Co/Co_9_S_8_@NSC‐2,^[99]^ while weaker peaks at 29.8° and 52.1° are attributed to the (311) and (440) crystal faces of Co_9_S_8_, confirming the Co/Co_9_S_8_@NSC heterostructure formation. With increasing thiourea concentration (x=0.3, 0.5, 0.7, and 0.9), the product gradually transitions from Co/Co_9_S_8_@NSC to Co_9_S_8_@NSC. Transmission electron microscopy (TEM) (Figure 11c) shows Co/Co_9_S_8_@NSC‐2 composed of minute nanoparticles with a thin shell. High‐resolution TEM images (Figures 11d and e) reveal lattice spacings of 0.21 and 0.29 nm, corresponding to the (111) and (311) planes of Co and Co_9_S_8_. Defects in the carbon layer facilitate electron transfer, enhancing catalytic performance. A distinct interface between Co and Co_9_S_8_ confirms successful Co/Co_9_S_8_@NSC‐2 heterojunction formation. Elemental mapping (Figure 11f–g) demonstrates even distribution of Co, S, C, and N throughout the structure. In summary, synthesis of the Co/Co_9_S_8_ heterojunction encapsulated in Co/Co_9_S_8_@NSC‐2 was achieved, highlighting its efficiency as a bifunctional electrocatalyst for ZABs.^[100]^

### 2.6 Metal/Carbon‐Based Heterostructure

Highly conductive carbon materials such as CNTs, graphene, and porous carbon have been utilized to make the heterostructure between metal‐based materials and carbon‐based materials. These metal‐carbon based heterostructures induced by the introduction of carbonaceous materials are a good choice to increase the electrical conductivity and ions transmission rate of all cells, considering versatile porous structure and durable electrical properties. Also, cheapness and ease of preparation are the main considerations for numerous researchers in the selection of carbon materials.

**Figure 12:**
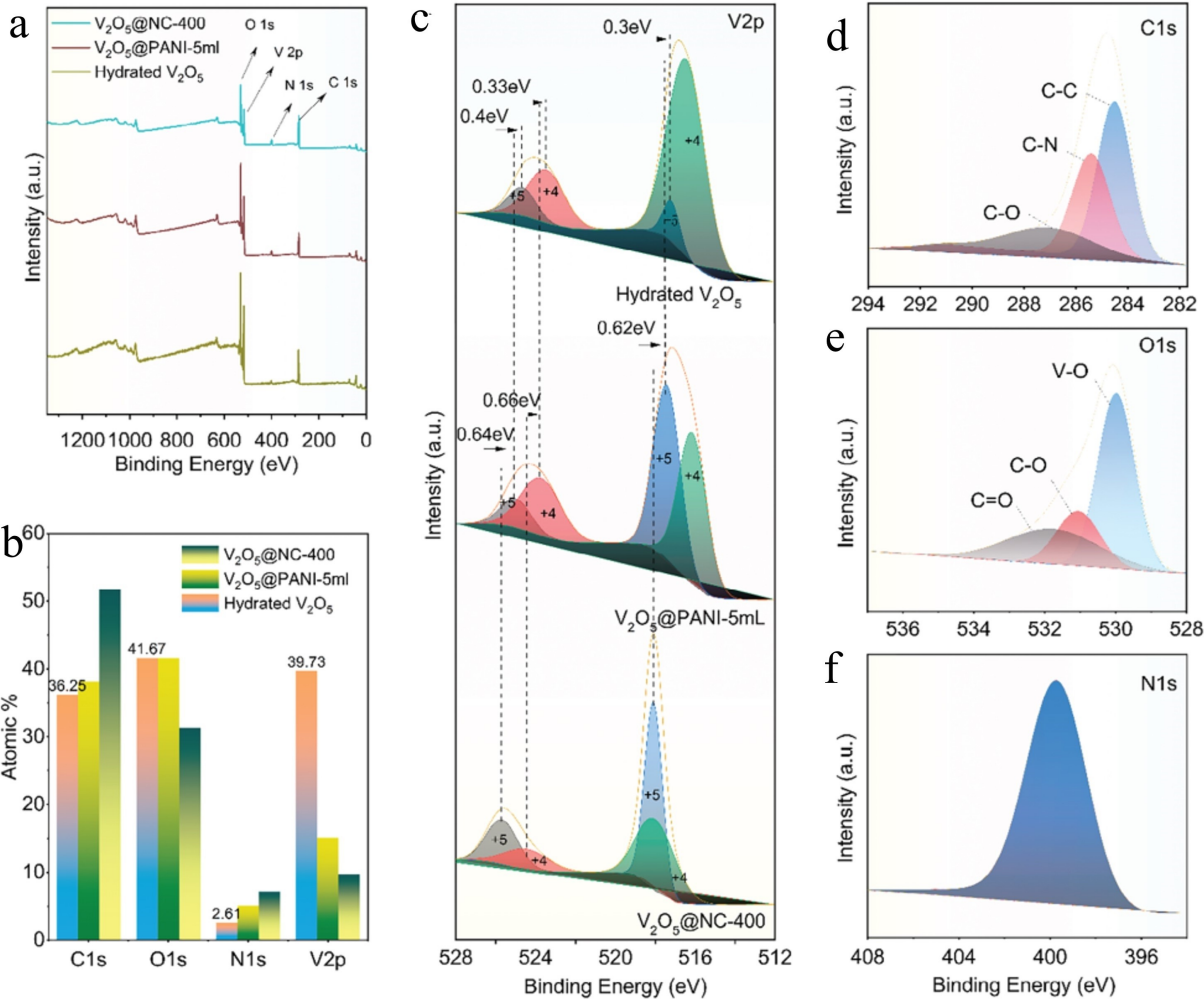
(a, b) Full XPS spectra of the samples and the as obtained atomic percentage results. (c–f) High‐resolution spectra for V 2p, C 1s, O 1s, and N 1s of the V_2_O_5_@NC‐400 heterostructure. Reproduced with permission from ref.^[101]^ Copyright 2023, WILEY‐VCH Verlag GmbH & Co. KGaA, Weinheim.

Najeeb ur Rehman Lashari et al. developed a straightforward method to synthesize the V_2_O_5_@NC heterostructure.^[101]^ Initially, hydrated layered V_2_O_5_ compounds were intercalated with aniline monomers to expand the interlayer spacing, followed by polymerization to form conductive polyaniline. Through a subsequent carbonization process, the electrical conductivity of the intercalated polymer layer was enhanced, resulting in the formation of a nitrogen‐doped carbon nanosheet layer. The synthesized V_2_O_5_/NC‐400 heterostructure was characterized using scanning electron microscopy (SEM) to reveal its layered structure, and transmission electron microscopy (TEM) for detailed examination. The interfacial interactions of hydrated V_2_O_5_, V_2_O_5_@PANI (5 mL), and V_2_O_5_@NC‐400 were investigated using X‐ray photoelectron spectroscopy (XPS) analysis, confirming the presence of V, O, and C elements in all samples.^[102]^ Compared to pure V_2_O_5_, an additional peak at 399.1 eV corresponding to the N 1s signal was observed in V_2_O_5_@PANI‐5 and V_2_O_5_@NC‐400, indicating the successful incorporation of PANI and N‐doped carbon into the layered V_2_O_5_ interlayer. The atomic percentage of elements (Figure 12b) showed gradual increases in the ratios of C and N atoms from hydrated V_2_O_5_ to V_2_O_5_@PANI‐5 mL and V_2_O_5_@NC‐400, attributed to PANI and its derivatives after carbonization. The V 2p spectra (Figure 12c) displayed peaks at 524.8 and 517.4 eV, indicating mixed valences of V^4+^ and V^5+^. After PANI intercalation and carbonization, shifts to higher binding energies in the V 2p spectra demonstrated interface interactions between V_2_O_5_ and the intercalated species, with an increased ratio of V5+ suggesting charge transfer to PANI and NC interlayers. The C 1s spectra of V_2_O_5_@NC‐400 (Figures 12d and e) revealed peaks at 284.5, 286.5, and 288.7 eV, corresponding to different carbon bonds of NC, indicating charge transfer pathways from V_2_O_5_ to NC through bridging O atoms in V_2_O_5_ nanosheets and C atoms in NC. The N 1s spectra (Figure 12f) exhibited a peak at 401.5 eV, indicating conversion of N atoms from PANI into pyrrolic‐N in NC.^[103]^ Nitrogen atoms’ affinity for zinc ions potentially enhances Zn ion diffusion within the heterostructures. In conclusion, V_2_O_5_@NC‐400 shows enhanced performance relative to pristine V_2_O_5_ due to improved electrical conductivity, expanded interlayer spacing, and increased affinity towards Zn ions facilitated by N dopants.

**Figure 13:**
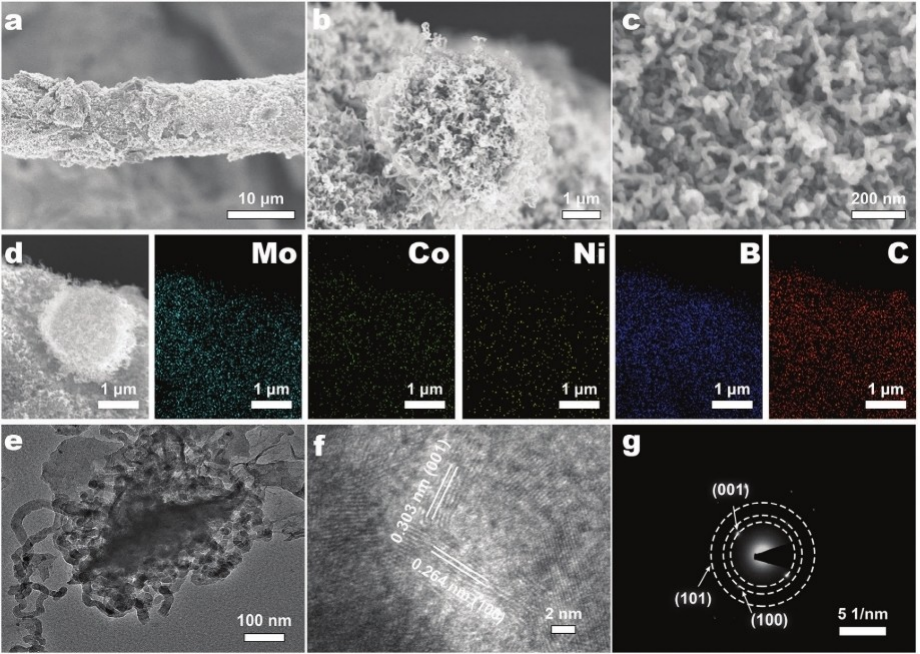
(a–c) SEM images of Co, Ni‐MoB_2_@CNT/CC; (d) the corresponding EDS elemental mapping images of Mo, Co, Ni, B, and C for Co, Ni‐MoB_2_@CNT/CC. (e) TEM image of Co, Ni‐MoB_2_@CNT/CC; (f) HRTEM image of Co, Ni‐MoB_2_@CNT/CC; (g) corresponding SAED patterns of Co, Ni‐MoB_2_@CNT/CC. Reproduced with permission from ref.^[104]^ Copyright 2023, WILEY‐VCH Verlag GmbH & Co. KGaA, Weinheim.

Sun et al. developed a novel method to create a carbon nanotube‐modified MoB_2_ catalyst on carbon cloth (Co, Ni‐MoB_2_@CNT/CC) using boronizing cobalt‐ and nickel‐containing polyoxometalate precursors.^[104]^ This approach leverages the heterogeneous structures of MoB_2_ and carbon nanotubes (CNTs) to provide numerous active sites for the hydrogen evolution reaction (HER).^[105]^ After high‐temperature boronizing and purification, NiMo_6_‐Co‐Complexes were clearly observed on the carbon cloth, roughening its fibers (Figure 13a). Detailed SEM images (Figures 13b and c) further reveal urchin‐like microspheres composed of nanotubes with a diameter of approximately 2 μm firmly attached to the carbon cloth fibers (Figure 13b). Cobalt presence aids in the decomposition of organic species into carbon during pyrolysis, resulting in the observed intricate nanotube structures where numerous curved nanotubes intertwine (Figure 13c). This unique morphology significantly increases the surface area of the electrocatalyst, facilitating ion adsorption and gas‐liquid transfer during catalytic processes. The presence of doped metals and organic species plays a crucial role in the formation of CNTs. Energy dispersive X‐ray (EDX) elemental mapping (Figure 13d) confirms a uniform distribution of Mo, Co, Ni, B, and C elements throughout the Co, Ni‐MoB_2_@CNT/CC structure. Transmission electron microscopy (TEM) images (Figure 13e1) show CNTs with an average diameter of 10–15 nm within Co, Ni‐MoB_2_@CNT/CC. High‐resolution TEM (HRTEM) images (Figure 13f) display lattice fringes corresponding to the (001) and (100) crystal planes of α‐MoB_2_, revealing significant dislocations and twin boundaries induced by Co/Ni doping, which are associated with the CNTs. The overlapping region of MoB_2_ and CNTs indicates the formation of a heterostructure, which enhances electron transfer between MoB_2_ and carbon for improved catalytic performance. Selected area electron diffraction (SAED) patterns (Figure 13g) further confirm the formation of Co, Ni‐MoB_2_@CNT/CC, showing discrete spots indexed to (001), (001), and (101) planes. Mo characteristic peaks in Co, Ni‐MoB_2_/CC and MoB_2_/CC exhibit negative shifts around 0.23 and 0.35 eV, respectively, indicating increased electron density near Mo atoms due to Co/Ni co‐doping and heterostructure formation. Defective CNTs also facilitate electron transfer and redistribution around active sites during alkaline electrocatalysis. Density functional theory (DFT) calculations support that the Co, Ni‐MoB_2_/CNT heterostructure effectively reduces water dissociation and optimizes hydrogen adsorption free energy, thereby enhancing HER activity.

Akkammagari Putta Rangappa et al. developed a method to synthesize COF/TiO_2_ heterostructures by hydrolyzing titanium isopropoxide (TTIP) onto the COF matrix.^[106]^ This approach involved inserting TiO_2_ nanodots into COF pores through ligand‐induced site‐specific nucleation. Coordinated bonds between metal precursors and bipyridyl units facilitated hydrolysis, yielding ultrasmall (approximately 1.82 nm) TiO_2_ nanodots regularly arranged within the COF matrix pores. They also prepared COF heterostructures based on Zn‐Ppy metalloporphyrin to explore the influence of TiO_2_ active site architecture on COF photoreduction properties.^[107]^ Experimental results indicated that site‐specific nucleation formed structurally regulated TiO_2_ active sites confined within the COF matrix without causing structural deformation. Enhanced COF‐TiO_2_ interactions via N−Ti−O bonds promoted effective electron transfer, thereby improving photocatalytic performance. Asmita Dutta employed a composite process involving the integration of WS_2_ nanotubes with an N‐doped carbon coating layer.^[108]^ Combining scanning electron microscopy (SEM) and transmission electron microscopy (TEM) measurements revealed that while the cylindrical structure of the WS_2_ nanotubes (NTs) remained unaltered, the carbon coating exhibited electronic interactions with the NTs. Additionally, mild CO_2_‐assisted oxidation resulted in thinning the carbon layer to 2–5 nm and forming defects on the carbon surface. Subsequent annealing of carbon‐coated nanotubes in an N_2_ environment led to composite recrystallization, creating a high concentration of active catalytic sites on the surface and enhancing electrical conductivity. Consequently, the HER catalytic activity of the composite significantly surpassed that of individually tested carbon and WS_2_ NTs. The effective reduction in overpotential was attributed to the synergistic effect of semiconductive WS_2_ nanomaterial and conductive carbon coating. The cost‐effective, scalable synthesis of WS_2_/C composites driven by carbon nanodots preserved the original NT structure, demonstrated efficient and stable HER electrocatalysis, and broadened potential applications in electrochemical catalysts and electronic devices. Yu‐Shuai Xu and colleagues developed an oxygen diffusion etching method to synthesize Mo_2_C nanocrystals covered by holey carbon sheaths. They achieved this by thermally condensing a mixture of melamine and ammonium molybdate in an N_2_ atmosphere at high temperatures.^[109]^ Subsequently, the researchers oxidized a portion of the Mo_2_C nanocrystals in air, forming MoO_3_. A mild etching process using a base solution removed the MoO_3_ components, creating voids between the non‐oxidized Mo_2_C layers (2D‐Mo_2_C). The enhanced electron enrichment of 2D‐M_2_C potentially enhances its chemical stability in reactions. The 2D‐Mo_2_C@NC catalyst exhibited consistent activity and maintained highly coupled interfacial structures throughout multiple cycles of recycling reactions.

## 3 Electrochemical Energy Applications

Doping with elements or compounds enhances the electrochemical activity of electrode materials, facilitating faster ion insertion and extraction processes.^[110]^ This enhancement improves the charge‐discharge rate and overall battery performance.^[111]^ Heterostructure plays a crucial role in optimizing material performance and is key design strategies for enhancing the electrochemical energy storage capabilities of materials.^[112]^ The presence of heterostructure increases the number of active sites on material surfaces or interfaces, providing additional energy storage locations.^[113]^ This boosts the material′s energy storage capacity and enhances cycling stability.^[114]^ Moreover, heterostructure effectively mitigates volume expansion and contraction during charge‐discharge cycles, thereby improving the structural stability and cycle life of the material.^[115]^

### 3.1 Supercapacitors

Supercapacitors, also called electrochemical capacitors, store energy through ion adsorption (electrochemical double‐layer capacitors) or fast surface redox reactions and ionic intercalation (pseudo‐capacitors). Typical materials include carbons, conducting polymers, transition metal oxides, dichalcogenides, nitrides, and carbides.^[116]^ However, their relatively low conductivity and energy density do not fully meet human needs.^[117]^ To address these challenges, researchers focus on developing electrode materials through metal doping and heterostructure engineering.^[118]^ Metal ion‐doped transition metal oxides are promising for high‐performance energy storage devices.^[119]^ For instance, chromium‐doped iron oxide (Cr‐doped α‐Fe_2_O_3_) nanosheets were synthesized using microwave assistance.^[120]^ These nanosheets provided a large reaction area and fast electron transport, enhancing electrochemical properties. This highlights Cr‐doped hematite as a potential electrode material for high‐performance supercapacitors, potentially addressing current energy demands. In another study, fluorine‐doped δ‐MnO_2_ for supercapacitors was prepared via chemical deposition.^[121]^ Varying doping amounts significantly increased δ‐MnO_2_′s specific surface area, enhancing structural stability and improving specific capacitance and cycling stability over pure phase samples. Density functional theory (DFT) calculations revealed slight displacement from the equilibrium Fermi energy, narrowing the forbidden bandwidth and enhancing electronic conductivity in doped MnO_2_ samples. Additionally, a synthesis strategy involved parallel production of phosphorus‐doped ZnCo_2_O_4_ (P‐ZnCo_2_O_4_@NCC) and nitrogen‐doped carbon (NC@NCC) using ZnCo‐metal‐organic frameworks (MOFs) on dopamine‐modified carbon cloth (NCC) as conductive substrates.^[122]^ These materials showed excellent cycling stability and flexibility under different bending conditions. Two hybrid supercapacitors connected in series were able to illuminate a red LED when bent to 180°, demonstrating practical potential for real‐world applications. These advancements underscore the potential of metal‐doped and heterostructure‐engineered materials in enhancing supercapacitor performance, addressing key challenges in energy storage technology.

**Figure 14:**
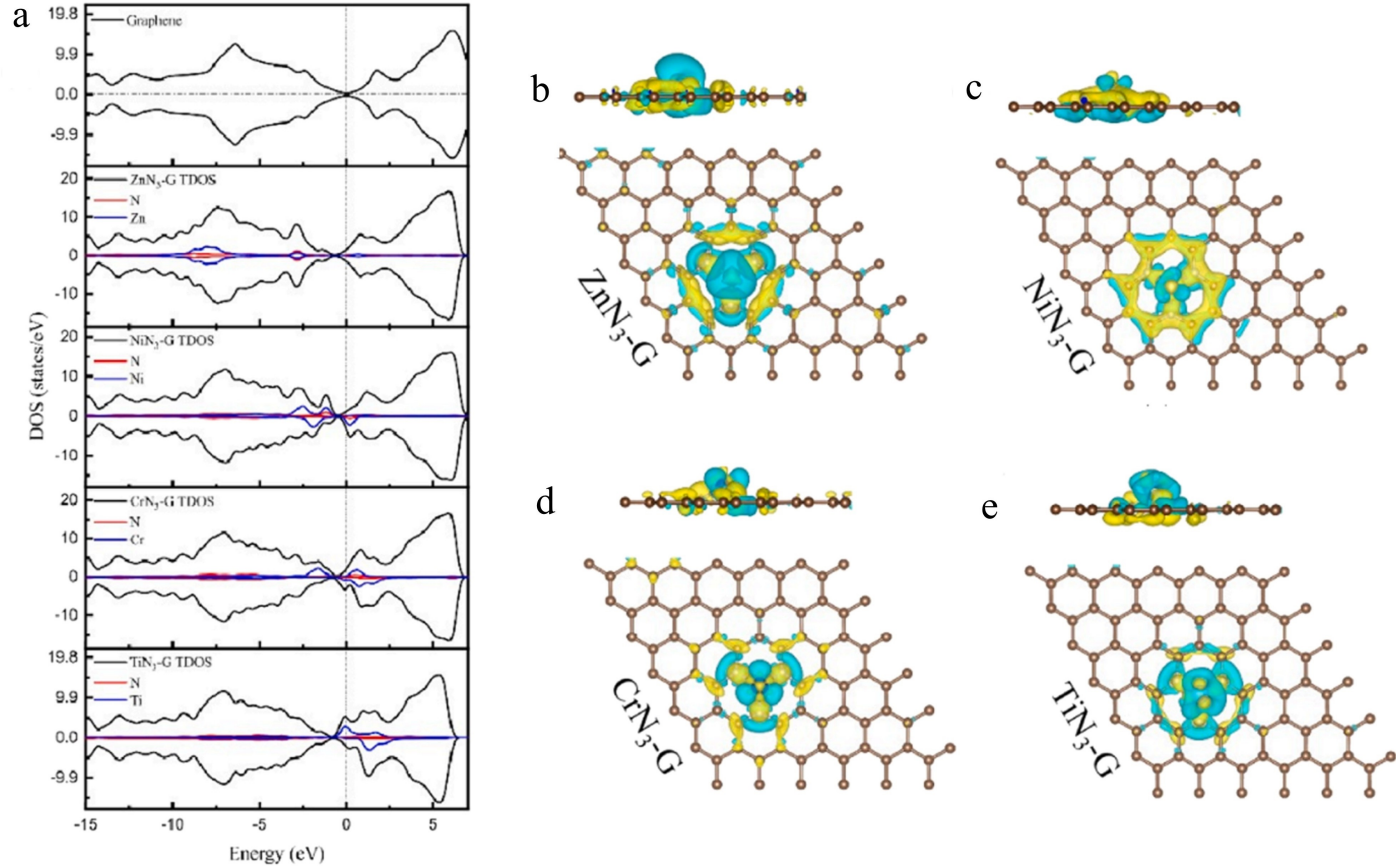
(a) Density of states of pristine graphene, ZnN_3_‐G, NiN_3_‐G, CrN_3_‐G and TiN_3_‐G, and (b–e) charge density difference iso‐surface mapping for ZnN_3_‐G, NiN_3_‐G, CrN_3_‐G and TiN_3_‐G. Yellow area represents charge accumulation, while blue area signifies zones of charge depletion. Reproduced with permission from ref.^[123]^ Copyright 2024, Elsevier B.V.

Cen et al. fabricated Co‐doped graphene systems by incorporating transition metal atoms (TM=Mn, Fe, Co, Ni, Cu, Zn, Cr, V, Ti) along with 3 nitrogen dopant atoms (TMN_3_‐G) (Figure 14).^[123]^ They utilized supercells based on a 5×5 graphene sheet, where each supercell substituted one carbon atom with a TM dopant atom surrounded by 3 N atoms. Various supercell sizes (2×2, 2×3, 3×3, 3×4, 4×4 atoms) were employed to explore different concentrations. To optimize atomic structure and calculate electronic properties, the Vienna Ab initio Simulation Program (VASP) with density functional theory (DFT) using the Perdew‐Burke‐Ernzerhof (PBE) parametrization and projected augmented wave (PAW) pseudopotentials were utilized. Analysis of charge density difference maps indicated electron accumulation around N atoms and depletion around metal atoms, indicating electron acceptance by N atoms and donation by metal atoms. Spin‐polarized calculations revealed asymmetric spin‐up and spin‐down states for NiN_3_‐G, CrN_3_‐G, and TiN_3_‐G, indicating spin polarization, while ZnN_3_‐G showed symmetric states, indicating a nonmagnetic state (Figure 14b–f). The Fermi level of CrN_3_‐G shifted up by 0.754 eV into the conduction band, creating local density of states (DOS) peaks contributed primarily by metal atom dopants, enhancing quantum capacitance (CQ) near the Fermi level (Figure 14a). Quantum capacitance exhibited a nearly linear increase within the voltage range, with higher values under positive bias compared to negative bias. The DOS profile showed varying contributions to capacitance at different potentials, resulting in an asymmetry in the CQ profile. CrN_3_‐G demonstrated the highest quantum capacitance due to its elevated DOS distribution near the Fermi level, particularly under positive voltage conditions. This suggests potential for CrN_3_‐G and similar materials in constructing asymmetric supercapacitor anodes.

**Figure 15:**
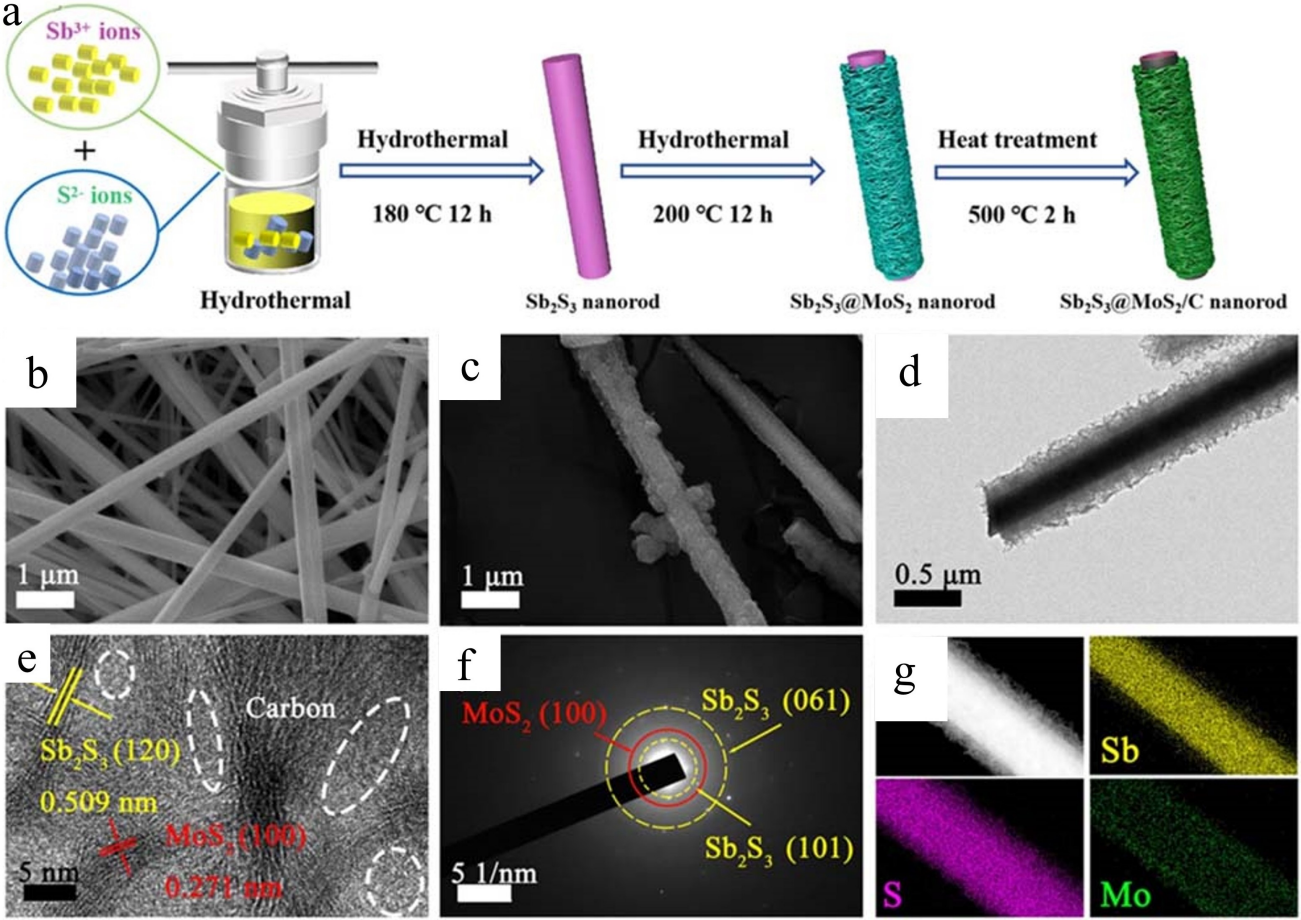
(a) Schematic illustration of the synthesis procedure of the Sb_2_S_3_@MoS_2_/C sample, (b) SEM images of Sb_2_S_3_, (c) SEM images of Sb_2_S_3_@MoS_2_/C, (d, e) TEM images of Sb_2_S_3_@MoS_2_/C, (f) SAED images of Sb_2_S_3_@MoS_2_/C, (g) EDS. Reproduced with permission from ref.^[124]^ Copyright 2024, Springer Nature.

Xue et al. developed a Sb_2_S_3_@MoS_2_/C heterostructure to enhance specific capacity and electrochemical performance compared to standalone Sb_2_S_3_ nanorods.^[124]^ The schematic in Figure 15a illustrates this heterostructure. Initially, sulfur‐based nanorods were synthesized via hydrothermal methods using three sulfur sources. Sb_2_S_3_ nanorods from CH_4_N_2_S showed a uniform distribution with an average diameter of 350 nm, as seen in SEM image (Figure 15b1617). Subsequently, Sb_2_S_3_@MoS_2_/C composites were synthesized via secondary hydrothermal reaction and calcination. SEM confirmed the nanorod‐like structure (Figure 15c), while TEM revealed a composite diameter of about 550 nm (Figure 15d). The MoS_2_ sheets intertwining on Sb_2_S_3_ rods form a network aiding electrolyte penetration and electron transfer.

Thanks to the MoS_2_ layer, the Sb_2_S_3_ nanorods feature a uniform core‐shell structure that effectively prevents volume expansion and maintains the nanostructure′s integrity and stability. In Figure 15e, clear lattice fringes with an interplanar spacing of 0.509 nm correspond to the (120) facet of Sb_2_S_3_, while the (100) facet of MoS_2_ shows a distinct interplanar distance of 0.271 nm. The lattice‐free edge region is composed of carbon. The selected‐area electron diffraction (SAED) pattern (Figure 15f) displays concentric circles and spots aligning with crystal facets of both MoS_2_ and Sb_2_S_3_, confirmed by matching diffraction rings. Additionally, weaker diffraction rings in MoS_2_ indicate lower crystallinity. Energy‐dispersive X‐ray spectroscopy (EDS) mapping (Figure 15g) verifies the core‐shell structure, with a dominant Sb signal in the core and uniform distribution of Mo and S signals throughout the rod‐like structure. Notably, the Mo signal is less pronounced due to its surface distribution on the Sb_2_S_3_ rod.

### 3.2 Alkaline Ion Battery

As battery technology advances, developing advanced electrode materials is crucial to meet the increasing demand for energy storage devices with higher energy and power densities.^[125]^ Various elements have been studied for lithium‐ion batteries (LIBs), sodium‐ion batteries (NIBs), and potassium‐ion batteries (KIBs).^[126]^ However, their low electrical conductivity, voltage hysteresis, and volume changes often lead to subpar electrochemical performance.^[127]^ Several strategies have been proposed to tackle these challenges. One effective approach involves constructing heterostructures that consist of multiple materials with different band gaps during charge and discharge processes, thereby creating built‐in electric fields at the interface.^[128]^ Additionally, metal‐doping has become essential for modifying the electronic and ionic states of active materials to enhance ion storage capabilities.^[129]^ Doped electrode materials exhibit altered intrinsic properties and crystal structures, impacting their charge state, bandgap, and stability.^[130]^ Hence, engineering heterostructures and metal‐doping are critical for advancing battery materials. These designs optimize electrochemical kinetics, boosting ion diffusion rates and electron transfer speeds, thus improving the overall performance of electrode materials in charge‐discharge cycles.

Liu et al. developed WS_2_/C hybrids using organic amine intercalation and in‐situ pyrolysis, resulting in porous WS_2_/C particles via dissolution and etching methods.^[131]^ The porous structure significantly increased the specific surface area, facilitating sodium ion insertion/extraction and enhancing reversible capacity. The carbon‐WS_2_ interface promoted rapid electron transfer between layers, leading to improved cycling stability and a reversible capacity of 346.3 mAh g^−1^ over 80 cycles at 100 mA g^−1^. This composite also demonstrated superior sodium storage performance and enhanced conductivity, offering insights for designing high‐capacity sodium‐ion and alkaline ion battery electrodes. Zhang et al. integrated ultrafine V_3_S_4_ nanocrystals with N‐doped carbon (N−C) through V−C bonds, embedded in a 3D porous structure to address volume changes and low conductivity issues.^[127]^ The nitrogen‐doped carbon coating provided dual pathways for electron/ion transport, ensuring a large specific surface area and structural integrity. Enhanced interface coupling improved electron transfer and structure stability, enhancing electrochemical performance. This approach could extend to other carbon‐based transition metal composites, improving energy storage applications by enhancing electrode material electrochemical activity, speeding ion processes, and boosting battery charge/discharge rates.

**Figure 16:**
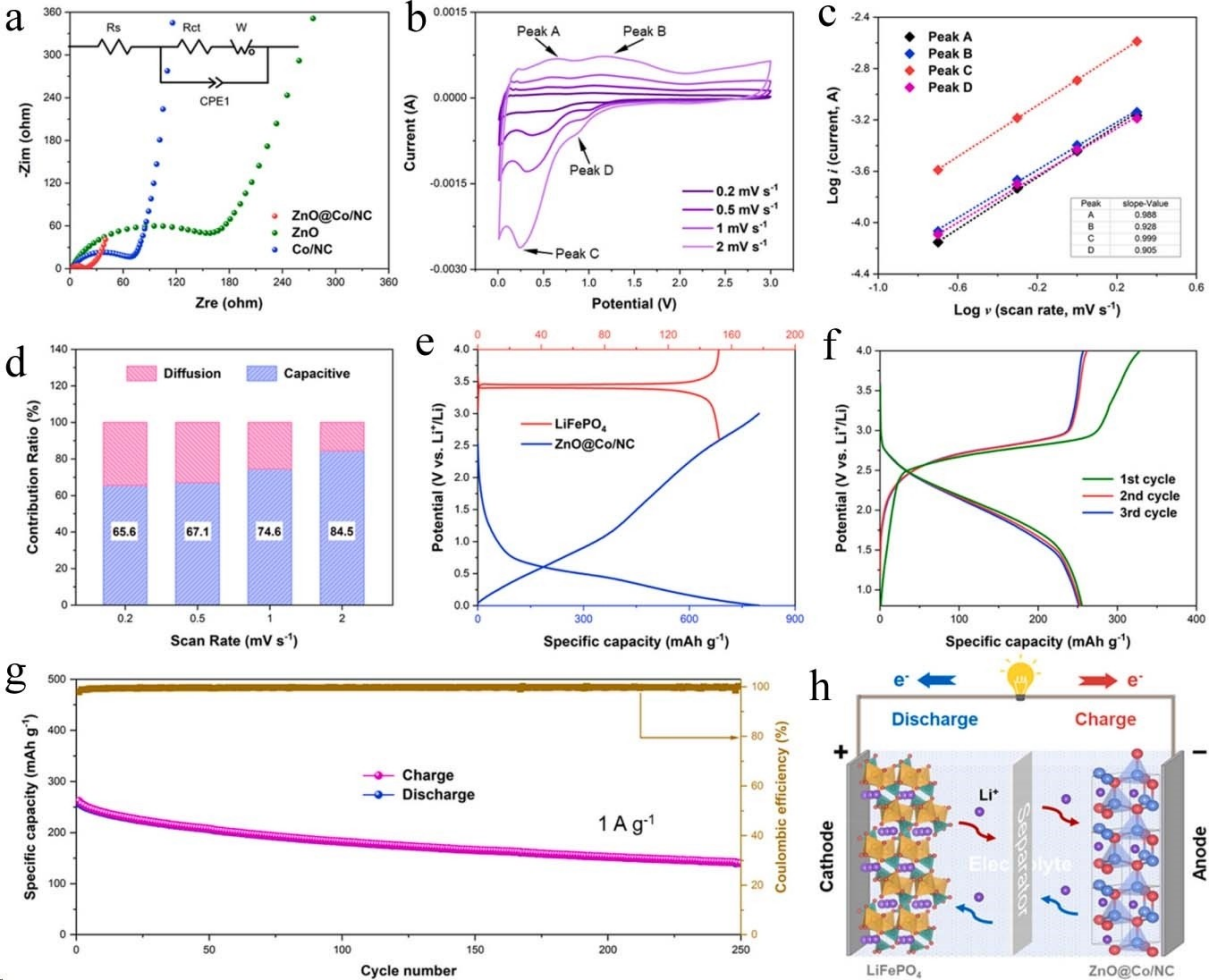
Lithium‐storage behaviors: (a) Nyquist plots of ZnO@Co/NC, ZnO and Co/NC electrodes (inset: selected equivalent circuit), (b) CV curves of ZnO@Co/NC at different scan rates, (c) Log (i) versus log (v) plots at cathodic and anodic peaks, (d) Contributions from capacitive capacities at different scan rates, (e) Charge/discharge voltage profles of Li//LiFePO_4_ and Li//ZnO@Co/NC in half cells, (f) The 1st, 2nd, 3rd charge/discharge curves of ZnO@Co/NC//LiFePO_4_ full cell, (g) Cycle performance of ZnO@Co/NC//LiFePO_4_ full cell, (h) Schematic illustration of the working principle of the full cell.^[129]^ Copyright 2023, Elsevier B.V.

Wang et al. synthesized ZnO@Co/NC core‐shell polyhedra using a zeolitic imidazolate framework as a precursor (Figure 16).^[129]^ This unique design forms a three‐dimensional conductive network that enhances the transfer and storage of Li^+^/K^+^ ions. It also mitigates the volume change of ZnO polyhedra during repeated lithium/potassium processes, thereby improving lithium and potassium storage performance. The ZnO@Co/NC anode maintained a capacity retention of 95 % with 458.3 mAh g^−1^ after 1000 cycles at 100 mA g^−1^ and 84 % retention with 181.5 mAh g^−1^ after the same cycles. This study provides insights into designing advanced electrodes for high‐performance LIBs and understanding the potassium storage behavior of ZnO‐based anode materials. Guan et al. synthesized a heterostructured cobalt sulfide/molybdenum disulfide‐based sodium‐ion battery anode material via hydrothermal and solid‐state sulfurization methods.^[126]^ Cyclic voltammetry (CV) spectra within a potential range of 0.3–3 V (vs Na+/Na) at a scan rate of 0.1 mV s^−1^ demonstrated excellent cycling stability. Initial cathodic sweeps showed peaks at 0.97, 0.75, and 0.46 V attributed to Na^+^ insertion (CoS to NaxCoS, MoS_2_ to NaxMoS_2_), shifting to higher voltages (1.34 V). Peaks at 0.75 V and 0.46 V indicated SEI layer formation and conversion processes (NaxCoS/NaxMoS_2_ to Co, Mo, and Na_2_S), shifting in subsequent cycles to 0.91 and 0.59 V. Anodic scans revealed peaks at 1.71 and 1.98 V, confirming CoS and MoS_2_ reformation. The third CV cycle mirrored the second, highlighting the high reversibility of the CoS/MoS_2_‐based anode. Galvanostatic charging/discharging curves at 0.1 A g−1 displayed three discharge plateaus (~1.4, ~0.92, ~0.61 V) and two charge plateaus (~1.68, ~2.0 V), consistent with CV observations.

The CoS/MoS_2_‐based anode initially exhibits a high discharging/charging capacity of 662.3/554.1 mA h g^−1^ and an initial Coulombic efficiency (CE) of 83.67 %, recovering to nearly 100 % in subsequent cycles. The irreversible capacity loss in the first cycle is mainly due to SEI layer formation. Cycling performance at 0.1 A g^−1^ shows gradual capacity decrease in the first 25 cycles, followed by slow recovery. Notably, at the 100th cycle, it achieves high capacities of 575.3/579 mA h g^−1^, retaining 108.4 % relative to the 2nd cycle. Comparatively, at 1 A g^−1^, the CoS/MoS_2_‐based anode demonstrates superior performance with a charge capacity of 510.9 mA h g^−1^ after 1000 cycles. In contrast, pure CoS decreases to 378.3 mA h g^−1^, significantly lower. Pure MoS_2_ starts low but stabilizes around cycle 650, reaching 453.6 mA h g^−1^ at cycle 1000. Long‐term stability at 2 A g^−1^ shows the CoS/MoS_2_ anode achieving 470.5 mA h g^−1^ with 91.1 % retention. Successful synthesis of flower‐like CoS/MoS_2_ via solid‐state sulfurization enhances stability and rate performance, attributed to its rational design enhancing kinetics and Na^+^ storage. Doping emerges as a critical strategy for enhancing battery performance, effectively improving overall performance and application capabilities by enhancing conductivity, boosting electrochemical activity, optimizing material stability, and increasing energy density.

**Figure 17:**
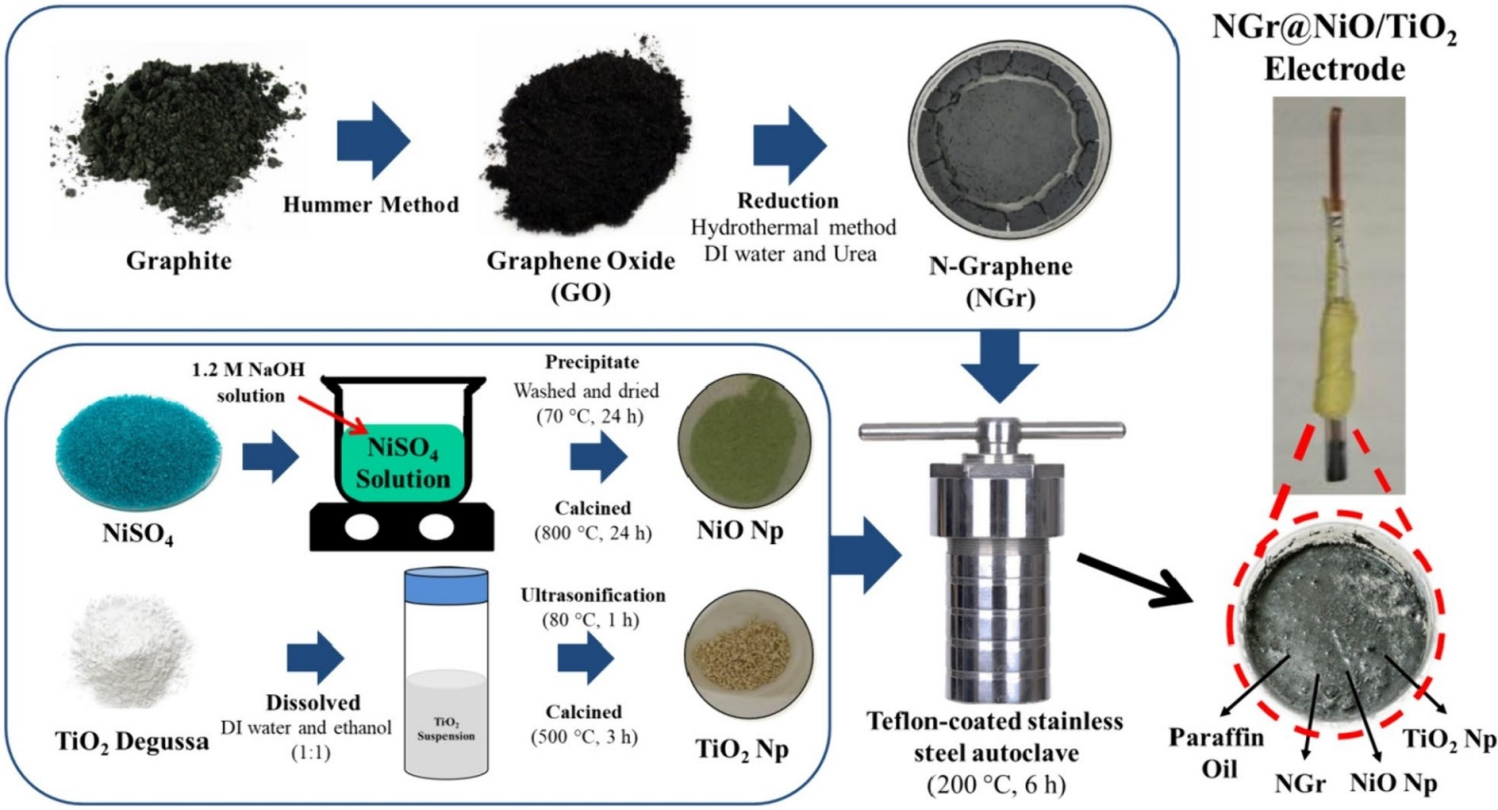
Scheme of synthesis procedure for the NGr@NiO/TiO_2_ hollow nanospheres electrode. Reproduced with permission from ref.^[130]^ Copyright 2024, Springer Nature.

Azis et al. explored a novel composite structure involving nitrogen‐doped graphene (NGr) and NiO/TiO_2_ hollow nanospheres, focusing on its potential for alkaline ion batteries like Li/Na/K batteries (Figure 17).^[130]^ The synthesized NGr@NiO/TiO_2_ nanocomposite displays distinct features characterized using appropriate techniques. NiO/TiO_2_ hollow nanospheres are evenly dispersed on NGr′s surface. The preparation involved dissolving NGr, NiO, and TiO_2_ in a specific mass ratio in deionized water. This suspension was transferred to a Teflon‐coated stainless‐steel autoclave and reacted at 200 °C, followed by cooling, washing, and drying. Subsequently, the product underwent annealing. Electrodes were made by mixing NGr@NiO/TiO_2_ with paraffin oil, stirring, placing the paste into a glass tube, smoothing the surface, and connecting it with a copper wire. The integration of NiO/TiO_2_ hollow nanospheres with NGr flakes *via* hydrothermal synthesis produced the NGr@NiO/TiO_2_ nanocomposite. FTIR characterization confirmed the material′s unique structure, crystallinity, morphology, and chemical bonds. Performance tests demonstrated that NGr combined with NiO/TiO_2_ nanospheres effectively reduces volume changes during charge‐discharge cycles, enhancing electrode stability and longevity.

### 3.3 Hydrogen Evolution Reaction (HER)

**Figure 18:**
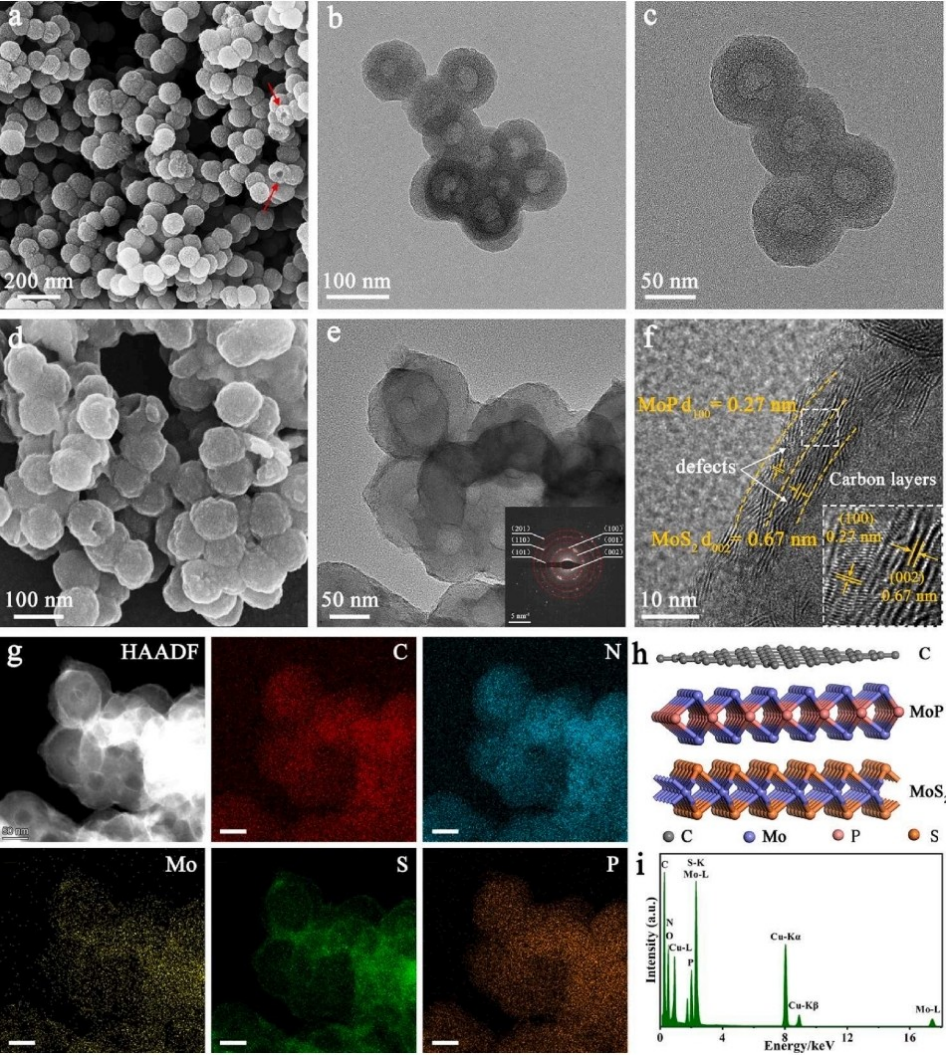
(a) SEM image of HCSs. (b, c) TEM images of HCSs. (d) SEM image of MoP‐MoS_2_/HCSs. (e) TEM image of MoP‐MoS_2_/HCSs. The inset shows the corresponding SAED pattern. (f) HRTEM imagine of MoP‐MoS_2_/HCSs. (g) HAADF‐TEM of MoP‐MoS_2_/HCSs and the corresponding elemental mapping (scale bars: 50 nm). (h) The structural model of the basic materials. (i) EDX elemental spectrum of MoP‐MoS_2_/HCSs. Reproduced with permission from ref.^[131]^ Copyright 2022, Elsevier B.V.

The hydrogen evolution reaction (HER) is essential in water electrolysis, converting renewable power into hydrogen. Electrocatalysts reduce HER overpotential, saving energy. Platinum‐based ones excel but are costly and scarce, hindering scalability. Efficient HER catalysts using abundant elements are crucial. Metal dopants create active sites, energy levels, and adjust hydrogen adsorption, enhancing conductivity and kinetics. Improving sluggish HER kinetics in alkaline environments demands optimizing hydrogen adsorption and water cleavage. Heterostructure catalysts with multifunctional components boost HER efficiency. Wang et al. developed triple‐shelled nanoreactors with ultrathin MoP‐MoS_2_ on N, P, S co‐doped hollow carbon spheres (MoP‐MoS_2_/HCSs).^[131]^ Poly(pyrrole‐co‐aniline) hollow nanospheres (PPCA) served as carbon precursors, with MoP‐MoS_2_ heterostructures formed via confined pyrolysis on PPCA. This structure exposed active sites, enhancing charge transfer. Nanoreactors efficiently catalyzed HER. TEM and HRTEM images confirmed interior hollow structures and ultrathin double‐shelled MoP‐MoS_2_ heterostructures on HCSs, validating composite formation. Additionally, the triple layers of HCSs, MoS_2_, and MoP formed a highly efficient pathway for electron transmission, significantly boosting the electrocatalytic performance of MoP‐MoS_2_/HCSs. High‐resolution TEM images revealed a distinct lattice spacing of 0.67 nm corresponding to the (002) crystal plane of MoS_2_, and an interplanar spacing of 0.27 nm attributed to the (100) facet of MoP. The close contact between the (002) facet of MoS_2_ and the (100) facet of MoP created a clear heterointerface, defining the MoP‐MoS_2_ heterostructure. Moreover, visible defects on the MoP‐MoS_2_ heterostructure indicated the presence of additional active sites. Analysis with HAADF imaging and EDX elemental mapping demonstrated the uniform distribution of carbon (C), nitrogen (N), molybdenum (Mo), phosphorus (P), and sulfur (S) throughout the hollow nanoreactor (Figure 18g). These elements were also evident in the EDX elemental spectrum (Figure 18i), confirming the effective integration of MoP‐MoS_2_ heterostructures on the HCSs. Consequently, the resulting triple‐shelled nanospheres, comprising N, P, and S co‐doped HCSs, MoP, and vacancy‐enriched MoS_2_, constituted a functional hollow nanoreactor (Figure 18h).

Li et al. ingeniously exploited the unique chemical properties of Ti_3_C_2_T_x_ MXene, where the exposed Ti atoms at the edges exhibit a positive charge, while the planar regions surrounded by surface functional groups (−OH, −F, and −O) display electronegativity.^[132]^ Leveraging this characteristic, they employed a sequential adsorption approach, beginning with the adsorption of MoO_4_^2−^ anions on the edges of MXenes, to vertically grow MoS_2_ flakes on the MXene edges, thereby fabricating lace‐like 1 T‐MoS_2_‐decorated MXenes (forming a MoS_2_/MXene interface).

**Figure 19:**
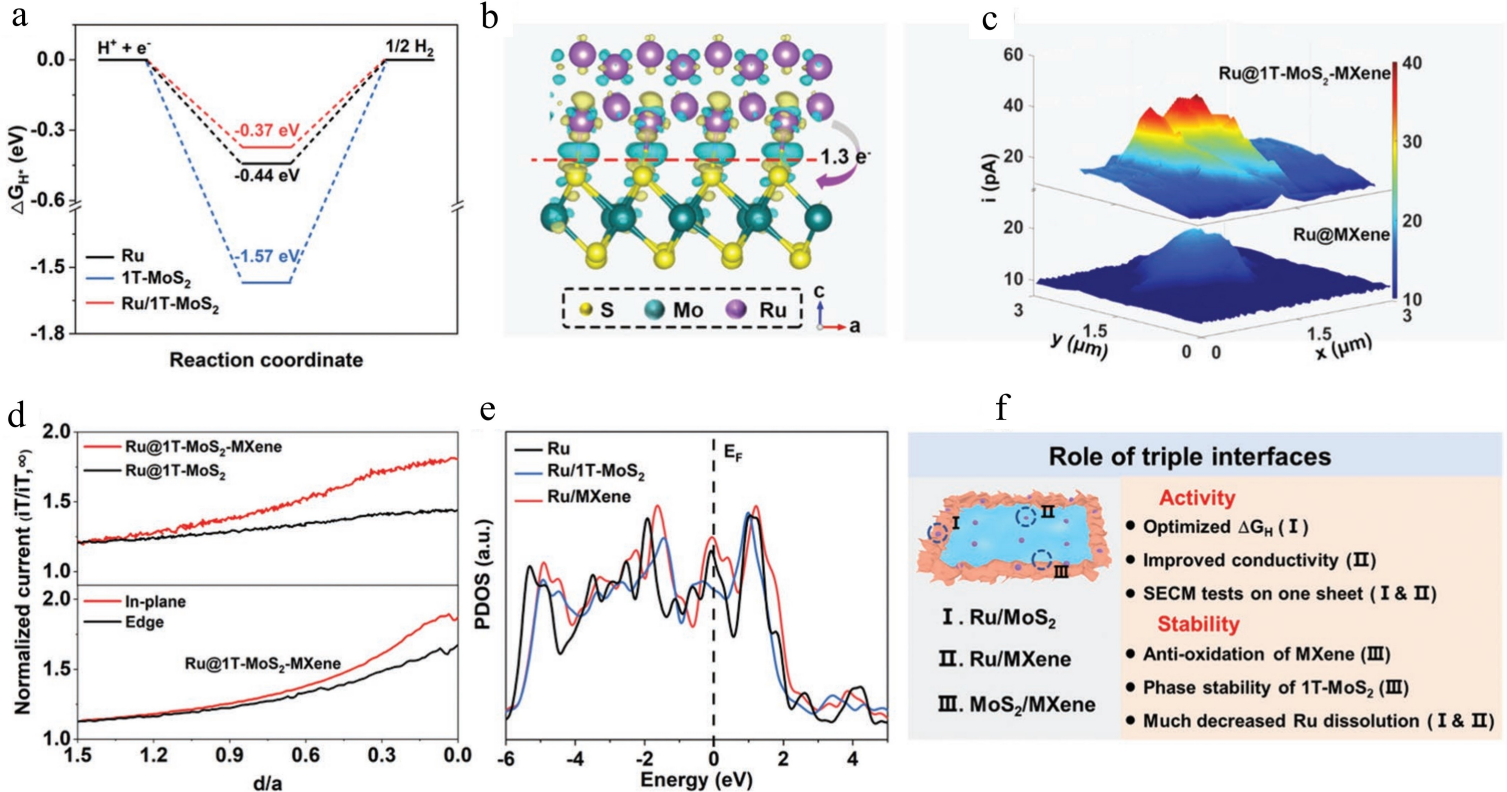
(a) Gibbs free energy for H* adsorption on different catalysts of Ru/ MoS_2_, 1 T‐MoS_2_, and pure Ru NPs. (b) Differential charge density distribution at interface of Ru/ MoS_2_. The charge accumulation and reduction are represented by yellow and green regions, respectively. (c) SG/TC SECM image of Ru@1 T‐MoS_2_‐MXene and Ru@MXene on HOPG for HER. The solution contained 5 mM HClO_4_, 1 mM Fc and 0.1 m KCl. ET=0.5 V, Es=−0.6 V versus Ag/AgCl, the tip radius is 17 nm, and the working distance is 40 nm. (d) Approach curves over Ru@1 T‐ MoS_2_‐MXene, Ru@1 T‐MoS_2_, and Ru@1 T‐MoS_2_‐MXene at different regions in solution containing 1 mM FcMeOH and 0.1 M KCl. ET=0.4 V versus Ag/AgCl and the substrates were unbiased. The tip radius is 26 nm. (e) Calculated DOS of Ru/MXene, Ru/MoS_2_ and pure Ru NPs. The black dashed line denotes the position of the Fermi level. (f) Advantages of the introduced three types of interfaces (Ru/MXene, MoS_2_/MXene, Ru/MoS_2_) for improved activity and stability. Reproduced with permission from ref.^[132]^ Copyright 2023, WILEY‐VCH Verlag GmbH & Co. KGaA, Weinheim.

The adsorption energy of H^*^ on the catalyst surface is crucial for evaluating hydrogen evolution reaction (HER) catalytic performance. Ideally, this energy should be close to zero. Figure 19a shows ΔG_H*_ values for Ru/MoS_2_, 1 T‐MoS_2_, and Ru, indicating that Ru/MoS_2_ exhibits superior catalytic performance compared to Ru and 1 T‐MoS_2_ alone. This suggests that the Ru/MoS_2_ interface optimizes H^*^ adsorption energy effectively (Figure 19a). Figure 19b depicts charge density differences at the Ru/MoS_2_ interface, revealing a transfer of approximately 1.3 electrons from Ru to MoS_2_, which weakens Ru′s H^*^ adsorption energy, as seen in the reduced ΔG_H*_ values for Ru/MoS_2_. To assess the impact of Ru/MoS_2_ and MoS_2_/MXene interfaces on HER activity, scanning electrochemical microscopy (SECM) in substrate generation/tip collection (SG/TC) mode was employed (Figure 19c). Hydrogen generation at the Ru@1 T‐MoS_2_‐MXene substrate was higher than at Ru@MXene, indicating 1 T‐MoS_2_′s significant influence on HER activity. These results, alongside those in Figures 19a–c, highlight the crucial role of the Ru/MoS_2_ interface in enhancing HER activity. Good conductivity is essential for catalytic materials, influencing their activity. SECM and density functional theory (DFT) calculations (Figure 19d, e) explored how introducing MXene and forming Ru/MXene and MoS_2_/MXene interfaces affect conductivity. The faster regeneration of the Fc mediator at the substrate surface is crucial. Comparing Ru@1 T‐MoS_2_‐MXene and Ru@1 T‐MoS_2_, the tip current increases more rapidly as d decreases, indicating faster proton transfer due to high conductivity MXenes. SECM experiments on a single Ru@1 T‐MoS_2_‐MXene sheet (Figure 19d) shows sharper current increase in the in‐plane vs. edge regions, highlighting the role of Ru/MXene in enhancing conductivity. First‐principles DFT calculations (Figure 19e) confirm higher electronic state density near EF for Ru/MXene vs. Ru/MoS_2_ and Ru, improving conductivity. Ru/MoS_2_ optimizes ΔG_H*_, while Ru/MXene enhances conductivity, boosting electrochemical activity (Figure 19f).

Deng et al. developed a straightforward method to synthesize Fe‐doped NiSe/Ni_3_Se_2_ heterojunctions on nickel foam (NF/NiSe/Ni_3_Se_2_‐Fe−X@t).^[133]^ They utilized SeO_2_ as the Se source and metal nitrates for Fe and Ni to minimize pollution. The optimized NF/NiSe/Ni_3_Se_2_‐Fe‐5@5 min heterojunction showed excellent electrocatalytic activity in alkaline media, achieving low overpotentials of 144 mV (HER) and 200 mV (OER) to reach a current density of 10 mA cm^−2^. Moreover, it demonstrated superior stability, lasting over 120 hours in water splitting operations. Jing et al. proposed a one‐step method for in situ nitridation and sulfidation.^[134]^ They synthesized phosphorus‐doped transition metal electrocatalysts using nitrogen, phosphorus, and sulfur sources from Saccharomyces cerevisiae via hydrothermal and high‐temperature carbonation processes. Starting with Co(OH)F NSs/CC, they fabricated heterojunction nanorods of phosphorus‐doped cobalt nitride/cobalt sulfide encapsulated by phosphorus and nitrogen co‐doped carbon nanoparticles on a carbon cloth substrate (P‐Co_5.47_ N/Co_9_S_8_@NPC NSs/CC). These catalysts integrated components for both OER and HER. Hu et al. developed a single‐step phosphating method to prepare a Ru‐doped WP/WP2 nanosheet heterojunction array on carbon cloth (Ru‐WP/WP_2_ NH/CC) using a Ru‐doped WO_3_ (RuWO_3_/CC) precursor.^[135]^ The nanosheet array morphology and tightly bound WP/WP_2_ heterojunction, along with Ru doping, synergistically enhanced HER performance. The Ru‐WP/WP_2_ NH/CC heterostructure exhibited HER performance comparable to Pt, with a low overpotential of around 58.0 mV at 10 mA cm^−2^ and a small Tafel slope of about 47.98 mV dec^−1^ in acidic electrolytes.

### 3.4 Oxygen Evolution Reaction (OER)

The oxygen evolution reaction (OER) constitutes another half‐reaction in water electrolysis.^[136]^ Noble metal oxides, such as RuO_2_ ^[62]^ and IrO_2_,^[138]^ are known as highly efficient OER electrocatalysts; however, they are limited by their high cost and scarcity in the Earth′s crust. Recently, various materials, including transition metal oxides^[139]^/hydroxides^[140]^ and carbon,^[141]^ have been explored for OER applications. Nonetheless, OER, being a four‐electron anode reaction, requires more energy to overcome its kinetic barrier compared to the hydrogen evolution reaction (HER). Therefore, besides selecting suitable materials, addressing fundamental challenges is crucial to achieve superior electrocatalysts. This involves developing more refined strategies, such as doping and heterostructure engineering. Li et al. employed iron doping to invert and enhance the interface electric field of heterojunctions, thereby shifting favorable binding sites from HER to OER.^[95]^ Self‐supported heterojunction catalysts (CoP@Ni_2_P/NF and Fe‐CoP@Fe‐Ni_2_P/NF) on nickel foam were synthesized with ease. The study illustrates that doping metallic elements is an effective strategy for modifying the interface electric field of heterojunction catalysts, thereby altering their catalytic properties.

**Figure 20:**
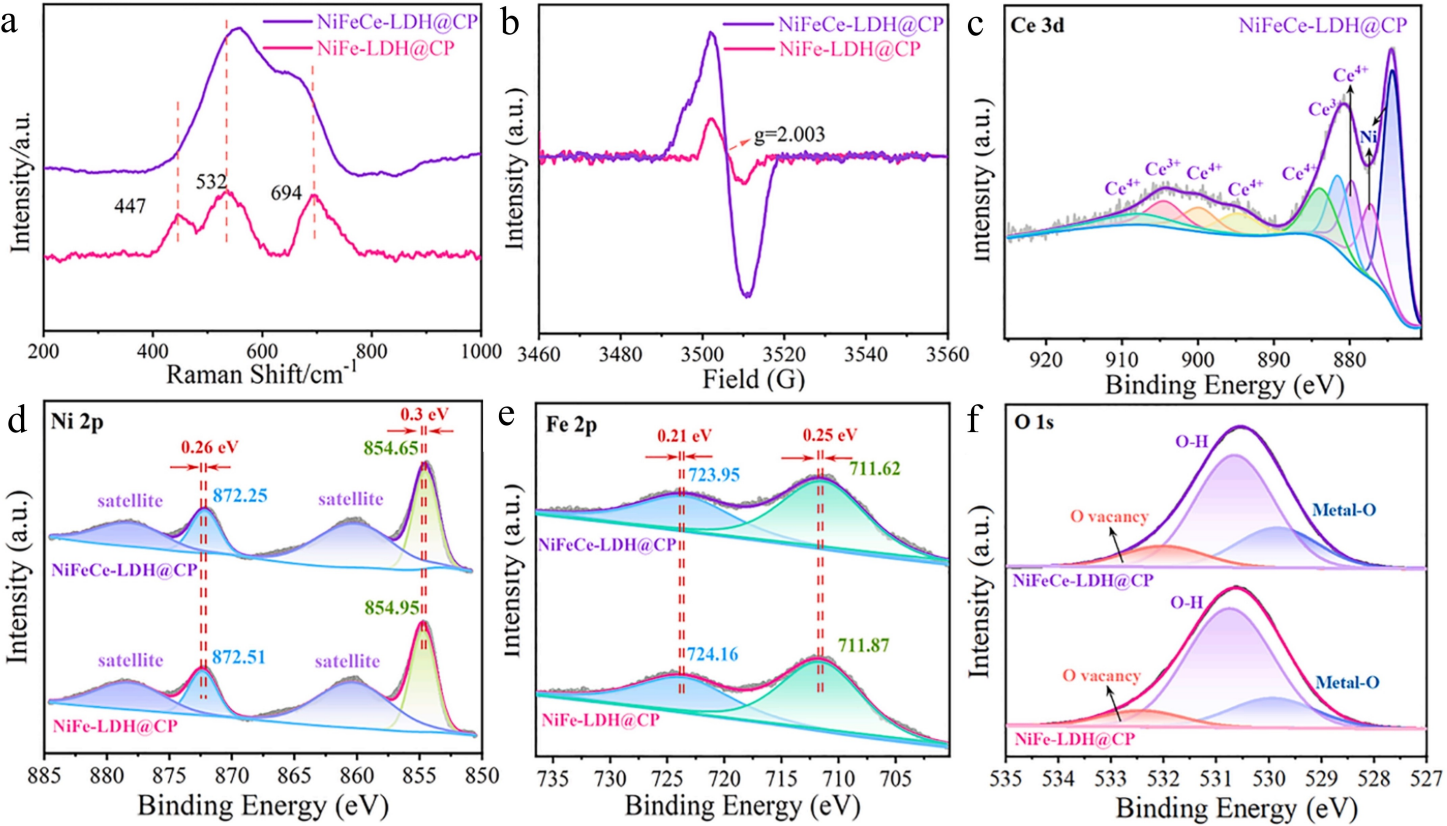
Raman spectra(a), EPR spectra(b), and XPS fine spectra of NiFeCe‐LDH@CP and NiFe‐LDH@CP for Ce 3d (c), Ni 2p(d), Fe 2p(e), O 1 s(f). Reproduced with permission from ref.^[142]^ Copyright 2023, Elsevier B.V.

Liao et al. proposed doping Ce into NiFe‐LDH to induce lattice distortion,^[142]^ enhancing surface area and oxygen vacancy formation, thus accelerating the Oxygen Evolution Reaction (OER) by modifying electronic structure and optimizing intermediate adsorption. Ni(NO_3_)_2_ ⋅ 6H_2_O, Fe(NO_3_)_3_ ⋅ 9H_2_O, and Ce(NO_3_)_3_ ⋅ 6H_2_O were dissolved in water and ethylene glycol, CO(NH_2_)_2_ added dropwise, then heated in a Teflon‐lined autoclave. NiFeCe‐LDH@CP showed stronger signals than NiFe‐LDH@CP in EPR and vibrational spectra confirmed LDH formation (Figure 20a). XPS spectra (Figure 20b) revealed Ce 3 d peaks at 923–907 eV (Ce^3+^) and 903–894 eV (Ce^4+^), enhancing redox capability (Figure 20c). Fe 2p XPS (Figure 20e) confirmed Fe^3+^ at 723.9 eV (Fe 2p_3/2_) and 711.6 eV (Fe 2p_1/2_); O 1s spectra (Figure 20f) showed more oxygen vacancies in NiFeCe‐LDH@CP, aligning with EPR analysis. Lattice distortion optimized Ni active sites, lowering energy barriers. Optimal NiFeCe‐LDH@CP exhibited excellent OER performance and low overpotentials. Zhao et al. proposed enhancing positive charge active sites and metal species by FeP_4_/CoP heterojunction formation via MIL‐88 A cation exchange, pyrolysis, and FeOOH promotion, improving oxygen intermediate adsorption and OER performance.^[143]^ The resulting FeP_4_/CoP/C catalyst achieved 258 mV overpotential for OER at 10 mA cm^−2^ and excellent stability over 52 hours at 20 mA cm^−2^.

Deng et al. assembled a series of Fe‐doped NiSe/Ni_3_Se_2_ heterojunctions on nickel foam using a one‐step electrodeposition method.^[133]^ By effectively accelerating electron transfer from Ni to Fe, the heterojunctions maintained their metallic state, with Fe^3+^ doping reducing the electron density of Ni^2+^. In terms of the Oxygen Evolution Reaction (OER), Ni served as the active site in NiSe/Ni_3_Se_2_, while Fe became the active site in NiSe/Ni_3_Se_2_‐Fe. In both materials, the rate‐determining step was the formation of the O→OOH intermediate, with the lower energy barrier (1.589 eV) after Fe doping contributing to enhanced OER activity. The interface synergistic effects of the heterojunction controlled the electronic structure and conductivity, beneficial for improving water splitting performance. During the OER process, iron became the active site, and its doping resulted in a smaller Gibbs free energy for the rate‐determining step (*O to *OOH), thereby imparting superior OER activity.

**Figure 21:**
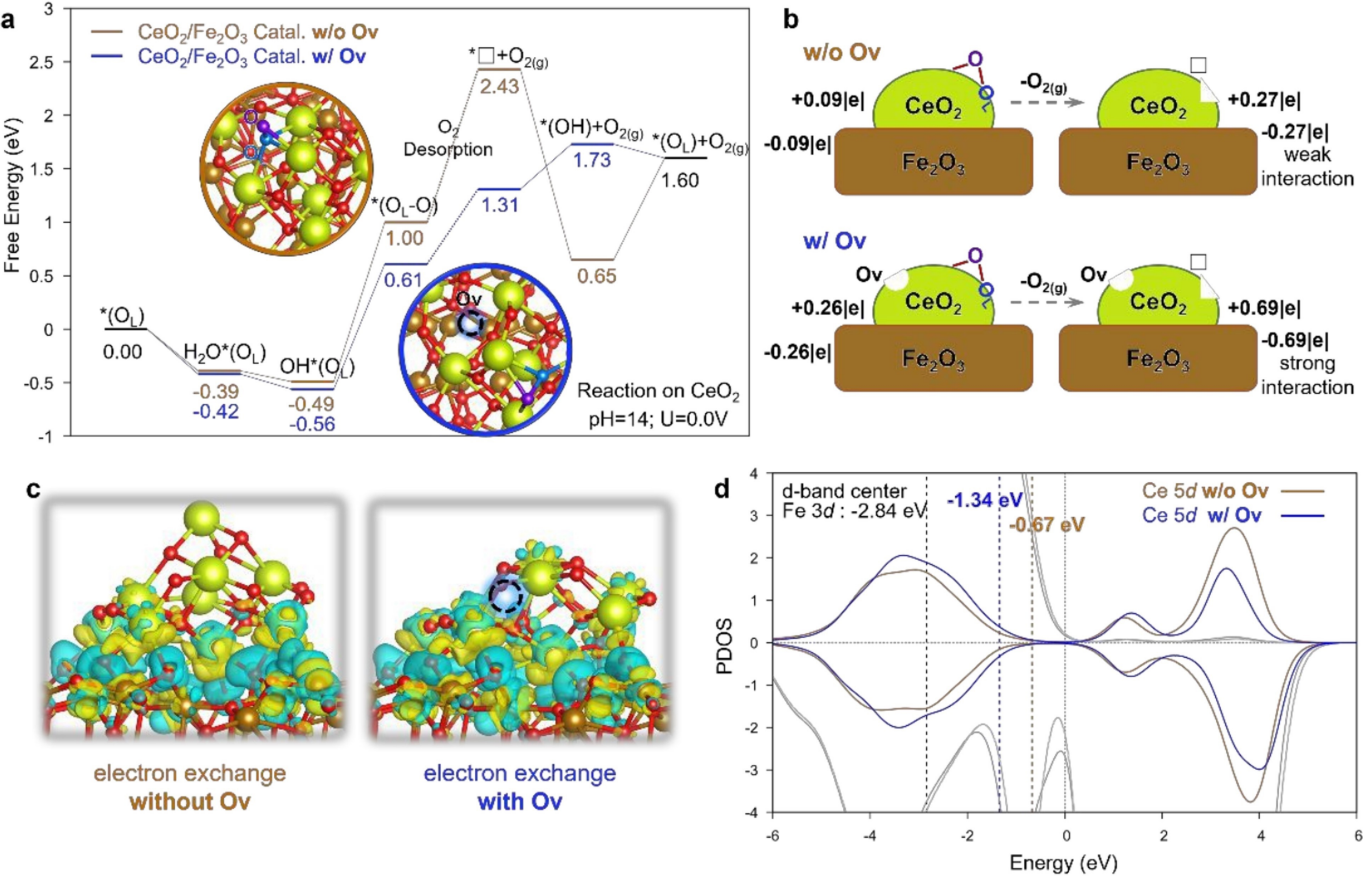
DFT calculations for LOM‐based OER catalyzed by the Fe_2_O_3_@CeO_2_‐O_V_ and Fe_2_O_3_@CeO_2_ catalysts. (a) free energy diagram for LOM based OER steps. The detailed 3D configurations are shown in Figure S34. Color code: Ce in yellow; Fe in brown; the O about to be removed in LOM in blue; the O from water in purple; the other O in red. The dotted lines represent the O_2_ desorption step. (b) Illustration of Bader charge changes during the O_2_ desorption step. (c) Charge density diagram showing the electron exchange between the CeO_2_ cluster and Fe_2_O_3_ support of the *ϒ intermediate after O_2_ desorption. Electron depletion and accumulation are depicted by yellow and blue areas, respectively. The isosurfaces are plotted at the value of ±0.03|e| Å^−3^. (d) Projected density of states (PDOS) of Ce 5d orbitals in the *ϒ intermediate of CeO_2_/Fe_2_O_3_ catalysts. The lighter and darker gray lines show the PDOS of surface Fe atoms with and without O_V_ on CeO_2_, respectively. The yellow and blue numbers show the d‐band‐center of Ce without and with O_V_. Reproduced with permission from ref.^[36]^ Copyright 2024, Royal Society of Chemistry.

Huang et al. utilized a self‐supported iron‐based metal‐organic framework (MOF) MIL‐53 nanosheet array, grown via solvothermal reaction in a mixture of Fe^3+^ in N, N‐dimethylformamide (DMF) and terephthalic acid (TPA) solution, on pre‐cleaned NF sheets.^[36]^ A secondary hydrothermal treatment of the MOF precursor in a Ce^3+^‐containing solution yielded the Fe_2_O_3_@CeO_2_ nano‐heterojunction electrode. The free energy diagram in Figure 21a illustrates the superior activity of the Fe_2_O_3_@CeO_2_ nano‐heterojunction electrode. Under standard alkaline conditions (pH=14, U=0 V), Fe_2_O_3_@CeO_2_‐O_V_ exhibits lower free energy compared to Fe_2_O_3_@CeO_2_, indicating higher activity. According to the OL−O formation step (OH*(OL) + OH^−^→(OL−O) + H_2_O + e^−^), the overpotential of the catalyst with OV decreases by 0.32 V upon O_V_ addition. Importantly, without O_V_, the Fe_2_O_3_@CeO_2_ catalyst exhibits a high internal energy free of 1.43 eV during O_2_ desorption ((OL−O)→*ϒ + O_2_(g)) (oxygen vacancy is abbreviated as *ϒ ), which significantly decreases to 0.70 eV with OV. To elucidate the underlying chemical principles, Bader charge analysis was conducted (Figure 21b). For Fe_2_O_3_@CeO_2_, CeO_2_ clusters without O_V_ show a slight positive charge (+0.09|e|) upon contact with the Fe_2_O_3_ surface, while the Fe_2_O_3_ surface exhibits a negative charge, indicating electron transfer from CeO_2_ to Fe_2_O_3_, consistent with experimental results. After O_2_ desorption, the positive charge of the CeO_2_ cluster increases by 0.18|e| (total increase of 0.27|e|). For Fe_2_O_3_@CeO_2_‐O_V_ with initial O_V_, CeO_2‐x_ clusters exhibit a higher positive charge (+0.26|e|), sharply increasing by 0.43|e| after O_2_ desorption.

Enhanced charge polarization underscores stronger interactions between CeO_2_ clusters and the Fe_2_O_3_ support. This phenomenon is evident in the charge density distribution of electron exchange at the heterojunction interface depicted in Figure 21c. For the *ϒ intermediate with O_V_, interactions between the clusters and support are notably robust, prompting CeO_2‐x_ clusters to subtly adjust their geometric configuration to tightly adhere to the support, analogous to “butter softening in a hot pan.” The Projection of Density of States (PDOS) and d‐band centers in Figure 21d further elucidate the impact of these strong cluster/support interactions. The results indicate that iron doping enhances the carrier density and intrinsic conductivity of the catalyst. This study offers crucial insights into optimizing the interface effects of electrocatalysts through element doping.

### 3.5 Oxygen Reduction Reaction (ORR)

The oxygen reduction reaction (ORR) plays a crucial role as a cathodic reaction in electrochemical energy storage devices like fuel cells and metal‐air batteries.^[144]^ A gradient doping strategy can be employed to establish a directional electrostatic potential difference, which enhances ORR catalytic activity significantly. Wang et al. pioneered the design and synthesis of ZnO doped with ultra‐low levels of iron (~0.15 %) as an exceptional 2e ORR catalyst (FeM‐ZnO) using a simple solution combustion method.^[146]^ FeM−ZnO exhibits superior ORR activity and selectivity by optimizing interfacial electron transfer kinetics, surpassing most recently reported metal oxide catalysts. Remarkably, FeM−ZnO demonstrates excellent stability withstanding 500 hours of continuous operation, marking the best 2e‐ORR stability among all metal oxide catalysts. Experimental and theoretical calculations illustrate that trace amounts of Fe accelerate electron transport kinetics and modulate the electronic properties of the catalyst bulk, ensuring optimal oxygen affinity and ^*^OOH adsorption/desorption capacity. This approach offers a straightforward yet effective strategy for modulating electron transport dynamics to regulate ORR selectivity and stability. Jiang et al., based on interface engineering concepts, utilized a direct ZIF‐67‐mediated etch‐anchored telluride strategy to fabricate hollow heterostructures consisting of highly dispersed CoTe_2_ within a mesoporous framework of N‐doped carbon nanoboxes and uniformly distributed NiTe_2_ on the outer surface (designated H‐CoTe_2_/NiTe_2_@NCBs).^[147]^ Compared with pure CoTe_2_ nanoboxes, the H‐CoTe_2_/NiTe_2_@NCBs heterostructure has excellent electrocatalytic performance and higher stability due to the significant synergistic effect between CoTe_2_ and NiTe_2_. Ting et al. investigated the doping of various transition metals (Ti, V, Cr, Mn, Co, and Cu) with differing t_2g_ and e_g_ occupancies into ferric nickel sulfide to produce ternary metal sulfides.^[148]^ They explored the effects of these metal dopants on electrical conductivity, electronic structure modification, and thermodynamic barriers. Both experimental and theoretical analyses demonstrated that Mn, Co, and Cu dopants enhance electrical conductivity. Ti, V, Mn, Co, and Cu dopants shift the D‐band center upwards, whereas Cr dopants shift the d of the Ni active site downwards, affecting the OER/ORR bifunctionality of ferro nickel sulfide.

**Figure 22:**
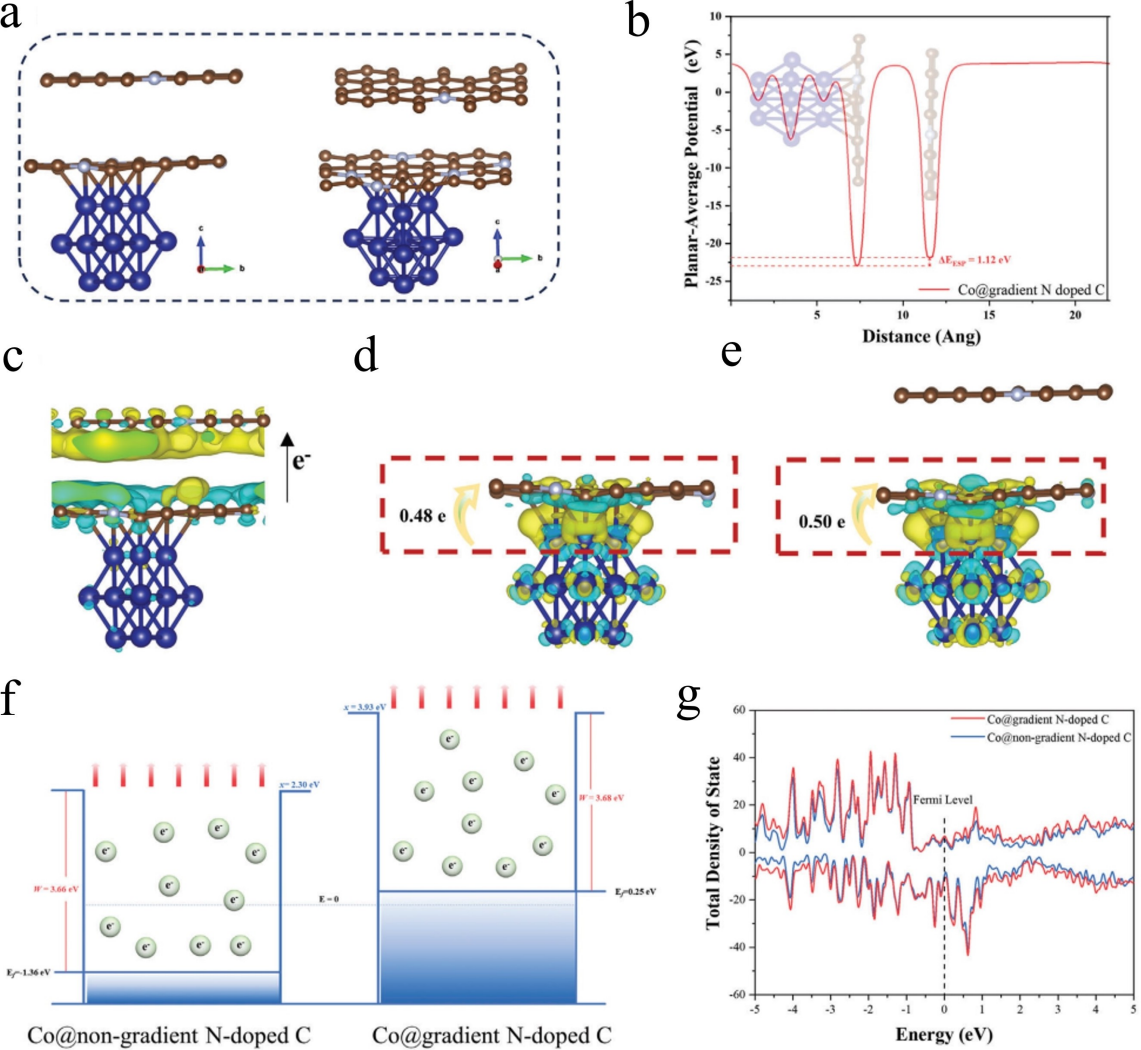
(a) Simulation model of Co@gradient N‐doped C (above: ND doped carbon layer, below: NR doped carbon layer). (b) The calculated electrostatic potential maps for Co@gradient N‐doped C. (c) Differential charge diagram of a gradient N doping model. Bader charge and differential charge density of (d) Co@non‐gradient N‐doped C, (e) Co@gradient N‐doped C (f) The WF of Co@gradient N‐doped C and Co@non‐gradient N‐doped C. (g) TDOS analysis of Co@gradient N‐doped C and Co@non‐gradient N‐doped C. Reproduced with permission from ref.^[149]^ Copyright 2024, WILEY‐VCH Verlag GmbH & Co. KGaA, Weinheim.

**Figure 23:**
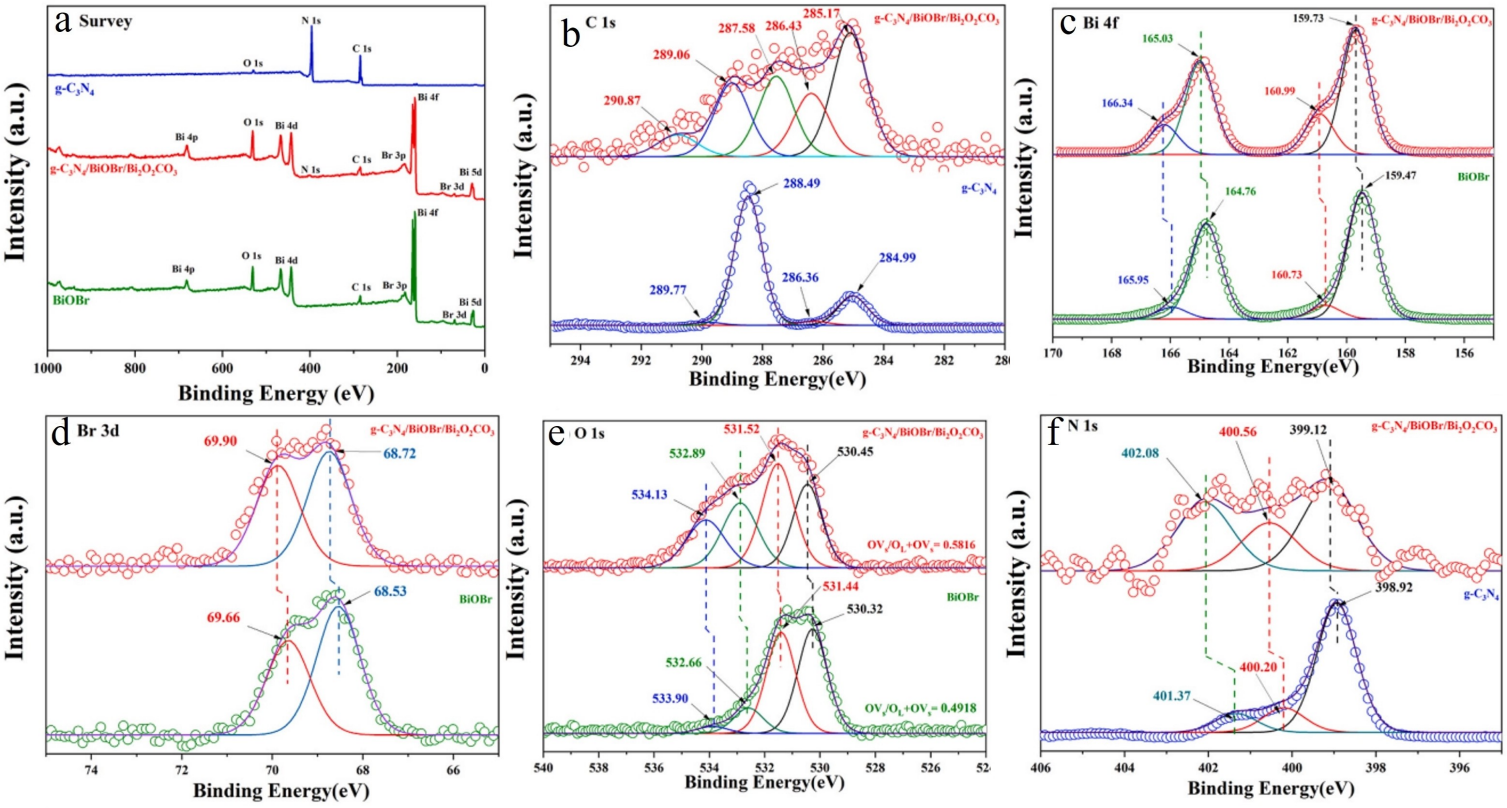
(a) Survey spectra of samples. High‐resolution spectra, (b) C 1s, (c) Bi 4 f, (d) Br 3d, (e) O 1s, (f) N 1s for g‐C_3_N_4_, BiOBr and g‐C_3_N_4_/BiOBr/Bi_2_O_2_CO_3_ ternary heterojunction. Reproduced with permission from ref.^[152]^ Copyright 2024, Elsevier B.V.

Similarly, Fu reported on a gradient nitrogen‐doped catalyst (Co@CNTs@NG), comprising nitrogen‐rich carbon nanotubes (CNTs) doped with cobalt nanoparticles (Co‐NPs) and graphene with nitrogen‐rich (NR) layers.^[149]^ In the gradient nitrogen‐doping model, a bilayer carbon substrate based on Co‐NPs was constructed: the upper layer composed of lower nitrogen‐doped carbon (ND carbon layer) and the lower layer of higher nitrogen‐doped carbon (NR carbon layer) (Figure 22a, 23). Calculations of the electrostatic potential gradient in the nitrogen‐doped model (Figure 22b) revealed a positive electrostatic potential difference (ΔEESP) of 1.12 eV along the z‐axis between the ND and NR layers, facilitating electron transfer from the NR to the ND layer. Differential charge density calculations indicated significant electron accumulation on the surface of the additional ND carbon layer, confirming that the electrostatic potential difference promotes electron transfer between the gradient nitrogen‐doped carbon layers (Figure 22c). Bader charge analysis further explored electron transfer between the carbon layers and the Co−NPs (Figures 22d and e).

Observations have indicated that the introduction of gradient nitrogen doping results in reduced charge transfer between the Co−NPs and the NR carbon layer. These findings suggest that the addition of the ND layer enhances variations in interlayer charge density, thereby influencing electron transfer between the NR layer and Co−NPs and ultimately optimizing the electron distribution on the surface of the NR layer. Furthermore, considering that the work function (WF) level determines the direction and feasibility of electron transfer, and that the electron transport capacity at the interface is crucial for the adsorption of intermediates, WF calculations were performed for two different models and simulated (Figure 22f). The results showed that Co@gradient‐N‐doped C exhibited a higher WF value (3.68 eV>3.66 eV) compared to Co@non‐gradient‐N‐doped C. An increase in WF implies a reduction in adsorption strength, which in turn reduces interaction with intermediates and promotes the desorption of intermediate species. Simultaneously, there was a significant upward shift in the material′s Fermi level, leading to a reduced barrier for electron filling in anti‐bonding orbitals, thereby facilitating the progression of electrochemical reactions. Total density of states (TDOS) calculations also indicated that Co@gradient‐N‐doped C had a higher density of states near the Fermi level compared to Co@non‐gradient‐N‐doped C, suggesting enhanced catalytic kinetics and superior electrical conductivity (Figure 22g). In summary, the electrostatic potential difference created by gradient nitrogen doping within the material promotes directional electron transfer, optimizing its DOS and WF, and thereby enhancing the ORR catalytic activity of Co@C materials.

Employing a synergistic heterojunction within the material emphasizes enhanced electrochemical performance and synergy at the interface, facilitating improved catalytic activity and stability for ORR applications.^[150]^ For example, Pattananuwat et al. developed a photoactive catalyst (g‐C_3_N_4_/BiOBr/Bi_2_O_2_CO_3_) for visible‐light‐driven photo‐assisted ORR via hydrothermal synthesis.^[152]^ XPS analysis investigated chemical states and surface compositions. Figure  23a showed XPS spectra of BiOBr, g‐C_3_N_4_, and the ternary heterojunction, confirming C, N, Bi, O, and Br elements. The C 1s spectrum in Figure  23b revealed g‐C_3_N_4_ with peaks at 289.77, 288.49, 286.36, and 284.99 eV (C−N−C, N−C−N, C−C, and C=C bonds). The g‐C_3_N_4_/BiOBr/Bi_2_O_2_CO_3_ spectrum exhibited peaks at 290.87, 289.06, 287.58, 286.43, and 285.17 eV (C−O−C, C−O, C=O, C−C, C−N bonds). In the Bi 4 f spectrum (Figure 23c), BiOBr peaks at 159.73 eV and 165.03 eV shifted to higher energies in the ternary heterojunction, indicating Bi−O bond formation. Additional peaks at 160.99 eV and 166.34 eV confirmed Bi−C bonds. Br 3 d spectrum (Figure 22d) showed Br peaks at 68.53 eV (3d_7/2_) and 69.66 eV (3d_5/2_) for BiOBr, slightly shifted in g‐C_3_N_4_/BiOBr/Bi_2_O_2_CO_3_. O 1s spectrum (Figure 23e) revealed BiOBr peaks at 530.32 eV (Bi−O), 531.44 eV, 532.66 eV, and 533.90 eV. The ternary heterojunction showed peaks, notably at 531.52 eV, indicating increased oxygen vacancies. N 1s spectrum (Figure 23f)  displayed peaks for g‐C_3_N_4_ and g‐C_3_N_4_/BiOBr/Bi_2_O_2_CO_3_, corresponding to different N bonding environments. These results suggest that the g‐C_3_N_4_/BiOBr/Bi_2_O_2_CO_3_ heterojunction alters core level spectra (O 1s, Bi 4 f, N 1s, Br 3d), enhancing electrochemical performance and stability for ORR applications.

## 4 Conclusions and Prospects

To date, electrode materials designed based on heterostructure and metal doping strategies have been extensively explored for electrochemical energy applications. This review provides a comprehensive summary of the latest advancements, covering synthesis, classification, and applications in electrochemical energy conversion and storage.

Firstly, we briefly introduce the classification and synthesis of mono‐/bimetallic doped engineering. Various metal elements (such as Fe, Co, Ni, V, W, La, Zr, Ln etc.) can be utilized as dopants to fabricate highly efficient materials through solvothermal methods, electrochemical deposition, chemical vapor deposition, and high‐temperature melting. Additionally, we summarize representative studies, further demonstrating the superior performance of materials with mono‐/bimetallic doped engineering for supercapacitors, batteries, HER, ORR, OER, and other applications. In conclusion, the advantages of mono‐/bimetallic doped can be categorized as follows: (i) metal doping engineering typically causes micromechanical disturbances, subtly distorting atomic arrangements and redistributing electron density; (ii) it can shift the d‐band center, tune orbitals, and induce more active sites, synergistically enhancing electrochemical performance; (iii) introducing vacancies after doping can reduce thermodynamic barriers in potential‐limiting steps of electrocatalysis. Furthermore, mono‐/bimetallic doping can lower overpotential, increase durability, and improve the rate performance of prepared materials.

Next, we particularly describe aspects of heterostructure engineering (e. g., amorphous‐crystalline, carbon‐metal, metal‐metal based heterostructures), which are constructed *via* solvothermal synthesis, electrodeposition, high‐temperature techniques, chemical vapor deposition, and co‐precipitation. Specific materials with heterostructure interfaces induce strong interactions that accumulate electrons at the interface, thereby tailoring electronic structures and adsorption abilities to deliver highly active electrodes for electrochemical devices. The enhanced activity of these materials is attributed to more active sites, superior conductivity, accelerated mass/charge transfer, and optimized intermediate adsorption—all associated with tuning the electronic structure and synergistic effects between heterostructured components. However, there are few reports dedicated to effectively controlling mono‐/bimetallic doped heterostructures through combined strategies and featured morphologies for achieving high‐efficiency and multifunctional electrochemical energy conversion and storage applications. Therefore, several challenges persist in the design and construction of mono‐/bimetallic doping/heterostructured materials, which we will discuss in the following sections:

- Developing cost‐efficient and scalable synthesis methods remains one of the primary challenges hindering the industrial application of metal‐doped and heterostructured materials. Although many of the materials discussed show excellent performance in electrochemical applications, most reported synthesis methods are limited to laboratory scales. Therefore, developing abundant and scalable preparation strategies is crucial to enable large‐scale commercial applications. Overcoming these challenges in the coming decade holds great potential for advancing the commercial viability of doping and heterostructured electrode materials.
- Designing high‐efficiency materials through mono‐/bimetallic doping and heterostructure engineering requires precise selection and control of dopant elements and concentrations to optimize structure, performance, and application suitability. It is crucial to meticulously manage the content of metal dopants and design various types of heterostructures to fabricate multifunctional electrode materials for diverse electrochemical applications. Introducing dopants into materials simultaneously alters several properties such as wettability, structural stability, surface chemistry, and morphology. Metal dopants can have both positive and negative impacts: (a) positively, they can act as unique accelerators by inducing subtle distortions in electron arrangements and redistributing electron density, thereby enhancing conductivity; (b) negatively, dopants may obstruct electronic transport channels due to irregular synergies between dopants and parent materials. Therefore, understanding the functional mechanisms of doping is crucial, and dopant‐induced changes must be considered when evaluating improvements in sample properties. Particularly, optimizing the synergy between these two engineering approaches is essential for developing high‐quality materials.
- Developing operando characterization methods is crucial for elucidating reaction mechanisms at each step when the material is in a reactive state. Combining the theoretical calculations (DFT), we will significantly understand these engineering strategies, thereby facilitating the design and construction of more efficient electrode materials.

## Conflict of Interests

The authors declare no competing interests.

## Biographical Information

*Dawei Chu is an Associate Professor at Huanghuai University. He received his Ph.D. degree in materials science and engineering from Harbin University of Science and Technology. Then, he moved to the National University of Singapore to continue his joint Ph.D. program as a sponsored exchange student. His research interests focus on the synthesis of advanced materials and their applications such as supercapacitors, overall water splitting, and capacitive deionization*.

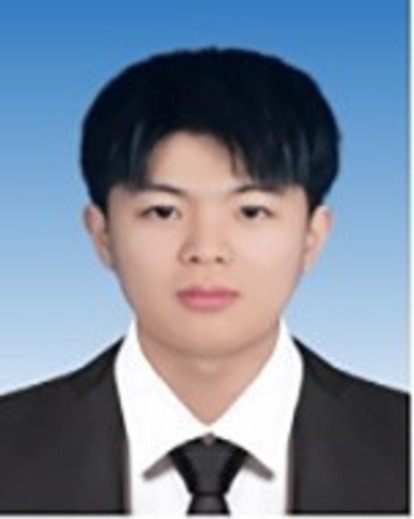

## Biographical Information

*Zhongwang Liang is a Lecturer at Huanghuai University. He received his master′s degree in materials science and engineering from University of Science and Technology of China. His research interests focus on the synthesis of advanced materials and their applications such as battery, supercapacitors, and overall water splitting*.

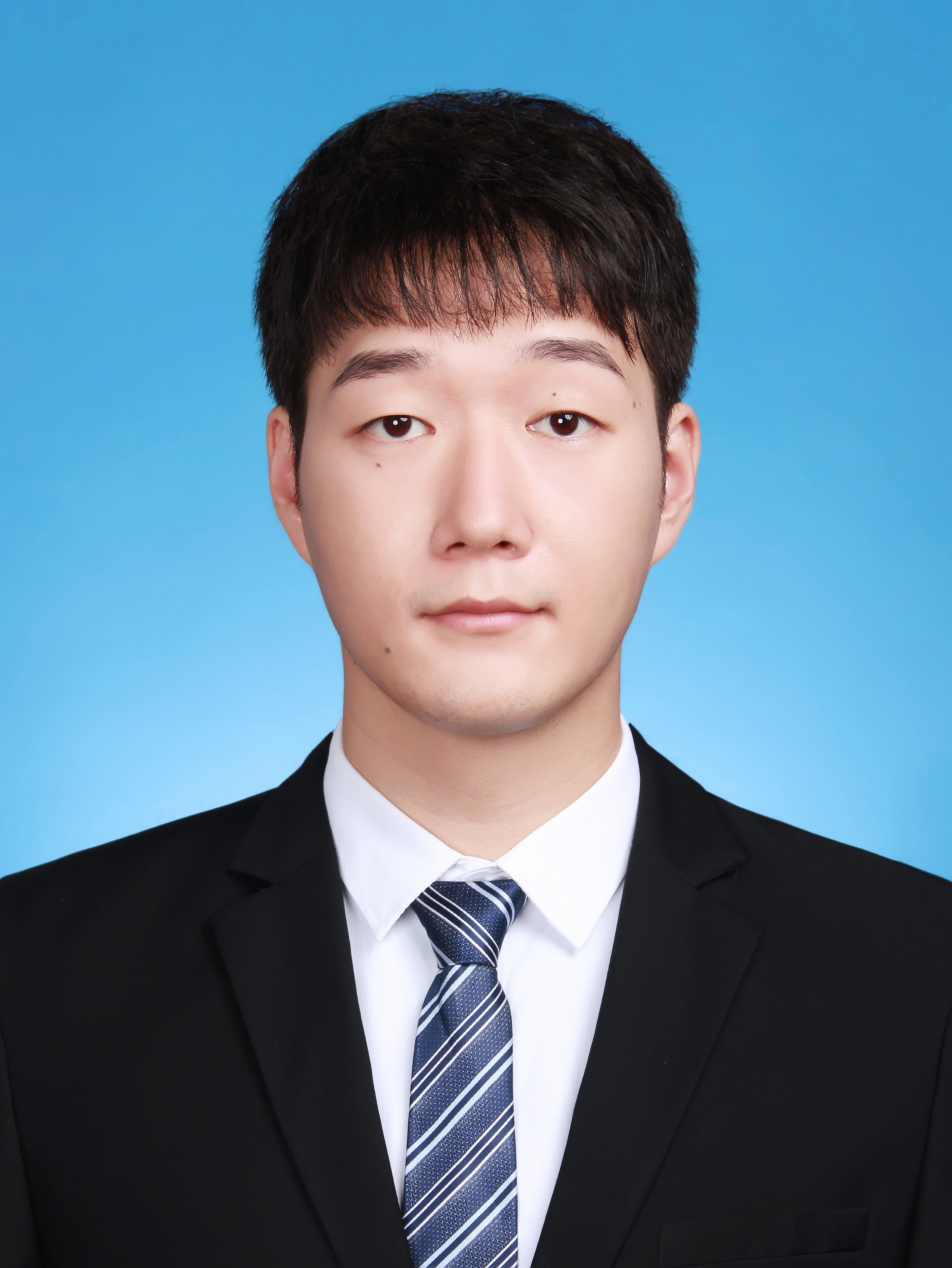

## Biographical Information

*Yi Cheng is an undergraduate student at Huanghuai University. His research interests focus on the synthesis of advanced materials and their application such as overall water splitting*.

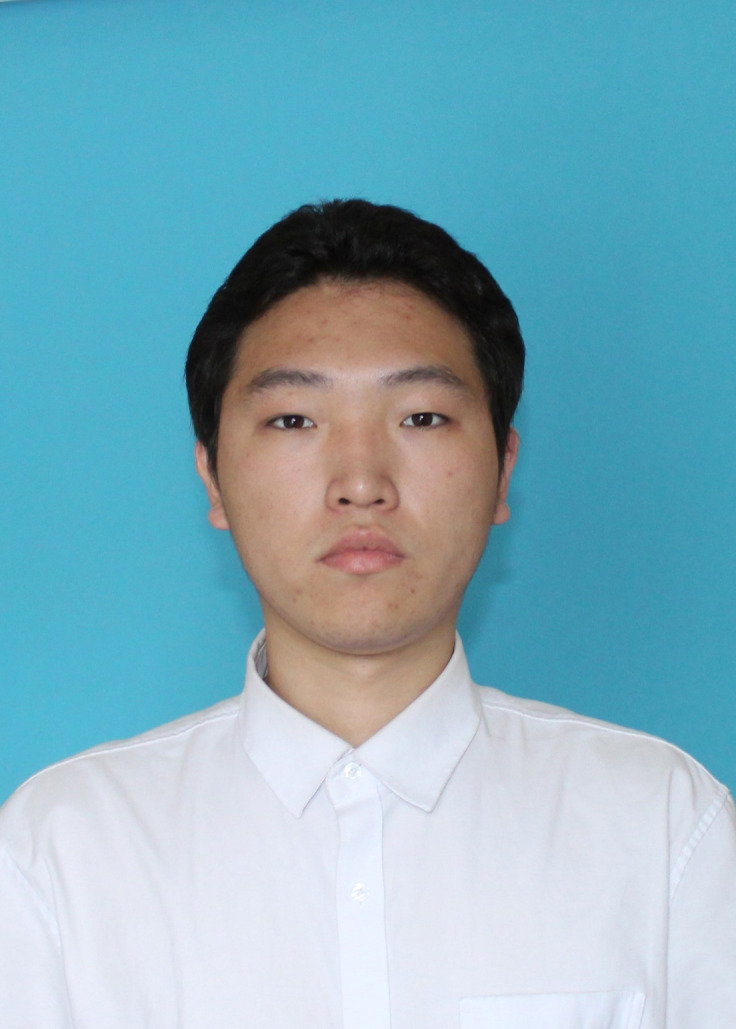

## Biographical Information

*Dong‐Feng Chai, received bachelor and master degree from Jiamusi University, and doctor degree from Harbin University of Science and Technology, postdoctoral research of Shanghai Jiao Tong University, a backbone teacher at Qiqihar University, associate professor, postgraduate supervisor, Heilongjiang Province Young Scientific and Technological Lift Talent*.

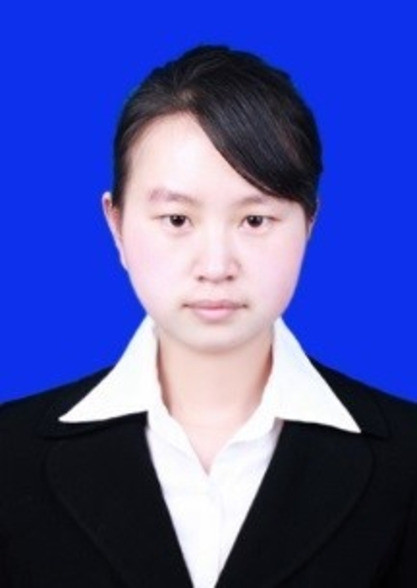

## Biographical Information

*Dr. Meijia Li is a Postdoctoral Research Associate at Pacific Northwest National Laboratory, specializing in the advancement of heterogeneous catalysis and advanced nanomaterials. With a Ph.D. in Materials Science and Engineering from Michigan Technological University, her focuses on the development of advanced catalysts such as boron nitride, high entropy metal oxides and their application for CO_2_ upgrading and syngas conversion. Dr. Li′s work aims to develop innovative catalysts for sustainable and efficient chemical processes*.